# Viruses in the tumor immune microenvironment: implications for immunotherapy

**DOI:** 10.3389/fimmu.2026.1958538

**Published:** 2026-10-21

**Authors:** Xuemin Chen, Ping Ni, Wei Li, Mingli Wu, Jun Li, Weinan Lu, Xuemei Li, Li Wang

**Affiliations:** 1 Department of Gastroenterology, Clinical Medical College and the First Affiliated Hospital of Chengdu Medical College, Chengdu, Sichuan, China; 2 Key Laboratory of Digestive Microecology Medicine of Sichuan Health Commission, Chengdu, Sichuan, China; 3 Key Laboratory of Digestive System Tumors and Microenvironment of Sichuan Provincial University, Chengdu, Sichuan, China

**Keywords:** cytokines, immune checkpoints, immune evasion, immunotherapy, oncolytic virus, tumor immune microenvironment, tumor-infiltrating lymphocytes, virus

## Abstract

The intricate interplay between viruses and the tumor immune microenvironment (TIME) plays a pivotal role in tumor initiation, progression, and response to immunotherapy. Viruses can directly infect tumor cells, modulate immune cell functions, and reshape cytokine networks, thereby influencing the composition and functionality of the TIME. This review systematically examines how viral infections impact TIME by altering tumor-infiltrating lymphocytes, cytokine profiles, and immune checkpoint expression. Additionally, the role of virus-associated antigens as immune targets and the mechanisms of virus-induced immune evasion that undermine immunotherapeutic efficacy are discussed. By integrating recent advances from single-cell omics, spatial transcriptomics, and clinical studies, this article aims to elucidate the core mechanisms underlying virus-TIME interactions. Furthermore, it highlights the therapeutic potential and challenges of virus-based immunotherapies, including oncolytic virotherapy and viral vector vaccines. This comprehensive overview provides a theoretical foundation for optimizing immunotherapeutic strategies that leverage viral components to enhance anti-tumor immunity and overcome immune resistance.

## 1 Introduction

The tumor immune microenvironment (TIME) constitutes a complex and dynamic ecosystem composed of tumor cells, various immune cell populations, stromal elements, cytokines, and extracellular matrix components. The immune status within the TIME can range from immunologically “cold,” characterized by low immune infiltration and suppressed immune activity, to “hot,” marked by robust immune cell presence and active antitumor responses. Understanding the factors that modulate the TIME is therefore essential for optimizing cancer immunotherapy strategies. Among these modulators, viruses represent a unique class of biological agents that can profoundly impact the TIME through diverse mechanisms. Certain oncogenic viruses, such as human papillomavirus (HPV), hepatitis B virus (HBV), and Epstein-Barr virus (EBV), contribute to tumorigenesis by establishing persistent infections that induce chronic inflammation and promote an immunosuppressive microenvironment conducive to tumor growth. Conversely, oncolytic viruses (OVs), including genetically engineered agents like talimogene laherparepvec (T-VEC), are designed to selectively infect and lyse tumor cells while simultaneously stimulating antitumor immunity, thereby reshaping the TIME from an immunosuppressive to an immunostimulatory state (1).

Oncogenic viruses drive tumor development by modulating immune checkpoints, altering cytokine profiles, and inducing metabolic and epigenetic changes that favor immune escape. In contrast, OVs exploit viral tropism and replication to directly kill cancer cells and release tumor-associated antigens, which in turn activate innate and adaptive immune responses. This immunogenic cell death (ICD) triggered by OVs leads to the recruitment and activation of dendritic cells (DCs), enhancement of antigen presentation, and priming of tumor-specific T cells, effectively converting “cold” tumors into “hot” ones with improved responsiveness to immunotherapies such as immune checkpoint inhibitors (ICIs) (2). The clinical success of T-VEC, an HSV-1-based OV expressing granulocyte-macrophage colony-stimulating factor (GM-CSF), in advanced melanoma exemplifies the therapeutic potential of harnessing viral agents to modulate the TIME and overcome tumor immune evasion (3).

Because the term “virus” in this setting covers agents with opposite biological roles, the categories used throughout this review are defined here. Oncogenic viruses are those that establish persistent natural infection and contribute to malignant transformation in a minority of infected hosts; they are commonly enumerated as seven agents, comprising HPV, HBV, hepatitis C virus (HCV), EBV, Kaposi sarcoma-associated herpesvirus (KSHV), human T-cell lymphotropic virus 1 and Merkel cell polyomavirus, with HIV acting indirectly through immunosuppression (4–6). The great majority of human viruses are not oncogenic, and the mechanisms described in Sections 2–4 refer specifically to persistent infection by these agents rather than to viral infection in general. Therapeutic viruses comprise replication-competent OVs, which lyse tumor cells and act as *in situ* adjuvants, and replication-deficient viral vectors, which serve as antigen or transgene delivery vehicles and are not oncolytic. OVs are themselves heterogeneous: platforms based on HSV-1, adenovirus, vaccinia virus, reovirus, measles virus and poliovirus differ in entry receptor, genome type, the innate sensors they engage, replication selectivity, transgene capacity and clinical maturity (7, 8), and these differences translate into divergent, occasionally opposite, effects on the same compartment of the TIME. Accordingly, each mechanism discussed below is attributed to a defined virus category and, where the evidence permits, to a named platform and experimental system. The same requirement applies to the strength of the evidence. Throughout this review the translational stage of each finding is stated where it is not evident from the text itself: results obtained in cell culture, in murine models, in non-human primates, in early-phase (phase I) trials, in randomized phase II or III trials, or in approved therapies are identified as such, and the clinical evidence column of **Table 1** records the stage reached by each strategy. Where only preclinical data exist, mechanistic plausibility is not presented as clinical efficacy.

**Table 1:** Virus- and viral-vector–based immunotherapeutic strategies targeting the TIME.

Strategy category	Representative viral platform	Armed transgene(s)	Mechanism of immune activation	Effect on the TIME	Target tumor(s)	Clinical evidence/stage	Combination partners	Limitations & challenges
**Unarmed oncolytic viruses** *(ICD induction)*	• Recombinant poliovirus • Myxoma virus • Reovirus • Vaccinia virus (VV) • Coxsackievirus • HSV-1	No exogenous payload — relies on intrinsic tumor-selective replication and lysis	• Selective infection and lysis of malignant cells • Induction of ICD • Calreticulin exposure; ATP, HMGB1 release • TBK1–IRF3 activation → sustained type I/III IFN (9) • PRR engagement (TLR3, RIG-I, cGAS–STING)	• DC recruitment and maturation • Th1-polarized CTL priming • IFN-γ–driven MHC-I upregulation on tumor cells (10) • Conversion of immunologically ‘cold’ to ‘hot’ TIME	• Melanoma • Glioblastoma • Multiple solid tumors	• Multiple Phase I/II trials • Mechanistic proof-of-concept established for several platforms	• Anti–PD-1/PD-L1 ICI • Stereotactic body radiotherapy (SBRT) (11)	• Clearance by neutralizing antibodies • Limited systemic delivery • Short viral persistence • Modest single-agent response rates
**Cytokine-armed OV** *(GM-CSF)*	T-VEC (talimogene laherparepvec) • HSV-1 backbone • ICP34.5 and ICP47 deletions • GM-CSF transgene	GM-CSF (granulocyte-macrophage colony-stimulating factor)	• Tumor-selective replication and oncolysis • Release of TAAs • GM-CSF–driven DC recruitment and maturation • DC cross-presentation → CD8^+^ T-cell priming • Systemic antitumor immunity (3, 12)	• Marked increase in local DC infiltration • Enhanced tumor-antigen presentation • Abscopal effect in non-injected lesions (13) • PD-L1 upregulation creates a target for ICI	• Advanced melanoma (FDA-approved) (3) • HNSCC (under investigation) (14) • Sarcoma (Phase II) (15)	• FDA-approved in melanoma • MASTERKEY-265 Phase III (T-VEC + pembrolizumab): ORR 48.6% vs 41.3%, DRR 42.2% vs 34.1%; PFS/OS endpoints not met (16) • Sarcoma Phase II: ORR 30% (15)	• Anti–PD-1 (pembrolizumab) — landmark combination in melanoma (17–19)	• Intratumoral delivery limits access to visceral metastases • Antiviral immunodominance may outcompete antitumor responses • Patient heterogeneity contributing to failure of survival endpoints
**Multi-cytokine/multiply-armed OV**	• OH2 (oHSV-2) (12) • Recombinant HSV • Oncolytic adenovirus • Oncolytic vaccinia	• IL-12 (potent Th1, IFN-γ inducer) • IL-15 (NK and memory T-cell support) • IL-7, IL-23, IL-9 (combinatorial) (20–22) • IL-36γ (23)	• Sustained local cytokine microenvironment • IL-12 drives Th1/CTL/IFN-γ axis • IL-15 sustains NK and CD8^+^ memory T cells • IL-36γ reduces MDSCs and M2 macrophages (23)	• Increased intratumoral CD4^+^/CD8^+^ T cells (21) • Expansion of NK and myeloid effectors • Reduction in Tregs and MDSCs • Elevated M1/M2 macrophage ratio	• Solid tumors (malignant ascites models) • Lymphoma • Glioma	• Robust preclinical efficacy • Selected platforms in early-phase clinical trials	• Anti–PD-1/PD-L1 ICI • Embelin (XIAP inhibitor) for OVV (24)	• Risk of systemic cytokine toxicity • Balancing immune activation versus tissue damage • Complex dose- and schedule-optimization
**ICI-armed OV** *(in situ checkpoint blockade)*	• Chimpanzee adenovirus (25) • Oncolytic vaccinia TG6050 (26) • HSV G47Δ (27) • YST-OVH (lentiviral) (28) • Recombinant influenza virus	• Full-length anti–PD-1 mAb or scFv • Anti–PD-L1 antibody • Anti–CTLA-4 mAb • Bispecific T-cell engagers (BiTEs)	• Tumor-selective infection • Local *in situ* expression of ICI antibody • High intratumoral concentrations with minimal systemic exposure • Concurrent oncolysis and checkpoint relief	• Increased CD8^+^ T-cell infiltration • Reduced Treg fraction • Elevated M1/M2 ratio (26) • Increased IFN-γ production • Conversion of immune-insusceptible to -susceptible tumors	• Pancreatic ductal adenocarcinoma (TG6050: IL-12 + anti–CTLA-4) (26) • Osteosarcoma (29) • Multiple solid tumors	• Preclinical superiority vs systemic ICI (25) • Favorable safety in non-human primates (YST-OVH) (28) • TILT-123 under clinical evaluation with anti–PD-1 (22)	• Systemic ICI to amplify abscopal responses • Anti–TIM-3/anti–TIGIT (29, 30) • Dual arming with IL-12 (31)	• Vector immunogenicity • Anti-vector neutralizing antibodies constrain re-dosing • Cargo capacity limits for multi-gene constructs
**Viral-vector vaccines** *(prophylactic + therapeutic)*	• Adenovirus (Ad5) • Chimeric Ad5.F35 (Ad5-neutralization evasion) (32) • ChAdOx1 (chimpanzee adenovirus) (33) • Modified vaccinia Ankara (MVA) • Lentiviral vectors	• HPV E6/E7 (cervical cancer) • EBV LMP2 (NPC) (34) • HBV HBsAg • Personalized neoantigens (NOUS-PEV: 60 neoantigens) (35) • Anti–CTLA-4 mAb gene (genetic adjuvant) (36)	• Efficient delivery of viral/tumor antigen genes • Broad CD8^+^ CTL and CD4^+^ helper responses • Generation of circulating and tissue-resident memory T (TRM) cells • Heterologous prime-boost (lentivirus prime → Ad/RNA boost) (37)	• Tumor-specific CTL expansion • Establishment of durable TRM pools • Enhanced DC activation • Intravenous boosting modulates myeloid compartment (38)	• NPC (LMP2 vaccine, Phase I validated) (34) • Cervical and HPV-associated cancers • Melanoma (personalized neoantigens) • Colorectal carcinoma	• Phase I (LMP2/NPC): induction of antigen-specific T cells (34) • Phase Ib (NOUS-PEV, melanoma): safety, immunogenicity, intratumoral neoantigen-specific T cells (35)	• Anti–CTLA-4 (early-dose priming) (39) • TLR7/8 agonist adjuvants (33) • Anti–PD-1 + Treg-depleting agents (40)	• Pre-existing neutralizing antibodies (especially anti-Ad5) • Autoimmune toxicity risk (notably with CTLA-4 blockade) • Manufacturing cost and turnaround for personalized regimens
**OV–nanovaccine platforms** *(no genetic engineering required)*	• Oncolytic adenovirus coated with tumor-antigen peptides (PeptiCRAd) (41) • Tumor-cell-membrane–coated OV nanovaccines (42)	• Externally coated tumor-antigen peptides • Tumor-membrane fragments displaying immunostimulatory ligands	• Personalization without viral genetic modification • Selective tumor targeting • ICD induction • Concurrent delivery of lytic and antigen signals	• Rapid induction of antigen-specific T-cell responses • Synergy with ICI blockade	• Ovarian carcinoma (preclinical) (42) • Multiple solid tumor models	• Preclinical evidence supporting personalized vaccine potential	• Immune checkpoint blockade	• Preclinical stage • Antigen-selection and personalized manufacturing constraints
**OV + systemic ICI combination**	• T-VEC + pembrolizumab (17–19) • Oncolytic adenovirus + anti–PD-1 (43) • OV + anti–CTLA-4 (HSV G47Δ) (27) • OV + anti–PD-L1 (camrelizumab) in advanced HCC (44)	Not applicable (sequential/concurrent agent administration)	• OV-induced ICD with DAMP and TAA release • Type I IFN–driven DC maturation and cross-priming • OV-driven PD-L1 upregulation creates an ICI target • ICI relieves T-cell exhaustion (12, 17, 18)	• ‘Cold-to-hot’ tumor conversion • Upregulation of CD8^+^ infiltration and granzyme B • Systemic immune activation in non-injected lesions • Coordinated upregulation of PD-1, PD-L1, CTLA-4 (13)	• Melanoma (primary evidence) • HNSCC • Sarcoma • HCC • ICI-refractory tumors	• MASTERKEY-265 Phase III (melanoma): higher ORR but PFS/OS not met (16) • HNSCC Phase Ib: tolerable; efficacy comparable to monotherapy (14) • Sarcoma Phase II: ORR 30% (15) • HCC + camrelizumab: encouraging response and tolerability (44)	This is itself the combination strategy • Further addition of anti–LAG-3/anti-CD39 • MDSC-depleting chemotherapy • Anti-angiogenic agents	• Antiviral vs antitumor immunity competition • Hepatic toxicity from repeat dosing in HCC (45) • Visceral metastasis accessibility • Patient and tumor heterogeneity
**Viral-vector vaccine + CTLA-4 blockade**	• Adenovirus-HPV E7 vaccine + ipilimumab (39) • Ad-9D9 (adenovirus co-expressing anti–CTLA-4 as genetic adjuvant) (36)	• HPV E7 or other viral/tumor antigens • Anti–CTLA-4 mAb gene (genetic adjuvant, co-delivered)	• Vaccine-driven expansion of antigen-specific CD8^+^ T cells • CTLA-4 blockade enhances CD80/CD86 costimulation • Intratumoral Treg depletion • Spatiotemporally synchronized co-delivery (36)	• Increased tumor CD8^+^ TRM formation • Durable maintenance of effector T-cell pools • Treg suppression	• HPV-associated malignancies (preclinical) (39) • Polyepitope neoantigen vaccine models	• Preclinical: significantly superior to monotherapy (39) • Co-delivery (Ad-9D9 same site) outperforms separate administration (36)	• Anti–PD-1 • Treg-depleting agents (near-complete tumor eradication) (40)	• Autoimmune toxicity (systemic CTLA-4 effects) • Critical timing and dosing optimization • Early-phase clinical translation only
**Next-generation engineered OVs** *(systemic delivery/immune evasion)*	• CD47-ectodomain–integrated OVs (46) • CD55-surface-modified OVs (complement evasion) (47) • PRV-LAV (attenuated pseudorabies virus vaccine) (48) • OVs arming anti–TIGIT/anti–TGF-β (27, 29)	• CD47 ectodomain (‘don’t-eat-me’ signal — evades MPS clearance) • CD55 (complement regulator) • Anti–TIGIT scFv • TGF-β trap/inhibitor	• Improved survival after intravenous administration • Evasion of neutralizing antibodies and mononuclear phagocyte clearance • PRV-LAV infects tumor cells via NRP1/EGFR → ER-stress–driven apoptosis (48) • Anti–TIGIT/TGF-β reverses local immunosuppression	• Enhanced immune-cell infiltration • Restoration of CD8^+^ T-cell effector function • Prolonged viral persistence despite neutralizing antibodies • Marked synergy with ICI	• Multiple solid tumors (beyond intratumoral-injection candidates) • Refractory or relapsed disease	• Predominantly preclinical evidence • Driving the paradigm shift from intratumoral to systemic OV delivery	• ICI • Small-molecule inhibitors • Anti-angiogenic agents • AI- and biomarker-guided personalization	• Early developmental stage • Engineering may compromise replicative competence • Complex regulatory pathway
**Epigenetic/metabolic modulation + OV**	• HSV or adenoviral OV • Combined HDAC inhibitors • Combined hypomethylating agents (decitabine) • Metabolic modulators (ROS scavengers, HIF-1α inhibitors)	No direct arming (combinatorial regimen)	• HDAC inhibitors reverse EBV/HPV-mediated MHC-I silencing (49, 50) • Hypomethylating agents restore IFN-γ signaling (EBVaGC) (51) • Metabolic modulation mitigates lactate-driven acidification of TIME • Synergy with OV-induced ICD	• Restored antigen presentation • NKG2D-ligand upregulation enhancing NK surveillance (52) • Reduced MDSC suppressive function • Improved TIL metabolic fitness	• EBV- and HPV-associated cancers • IDH-mutant glioma • HBV-associated HCC	• Strong preclinical synergy • Selected single-agent or combination regimens entering early-phase clinical trials	• ICI • Viral-vector vaccines	• Off-target effects of epigenetic modulators • Complex timing and dosing schedules • Lack of validated predictive biomarkers

BiTE, bispecific T-cell engager; ChAdOx1, chimpanzee adenovirus Oxford-1; CRT, calreticulin; DAMP, damage-associated molecular pattern; DC, dendritic cell; DRR, durable response rate; FDA, Food and Drug Administration; GM-CSF, granulocyte-macrophage colony-stimulating factor; HCC, hepatocellular carcinoma; HMGB1, high-mobility group box 1; HNSCC, head-and-neck squamous cell carcinoma; HSV, herpes simplex virus; ICD, immunogenic cell death; ICI, immune checkpoint inhibitor; ICP, infected-cell protein; IFN, interferon; mAb, monoclonal antibody; MDSC, myeloid-derived suppressor cell; MVA, modified vaccinia Ankara; NK, natural killer; NPC, nasopharyngeal carcinoma; ORR, objective response rate; OS, overall survival; OV, oncolytic virus; OVV, oncolytic vaccinia virus; PDAC, pancreatic ductal adenocarcinoma; PFS, progression-free survival; PRV-LAV, pseudorabies virus live attenuated vaccine; SBRT, stereotactic body radiotherapy; scFv, single-chain variable fragment; T-VEC, talimogene laherparepvec; TAA, tumor-associated antigen; TAM, tumor-associated macrophage; TIME, tumor immune microenvironment; TRM, tissue-resident memory T cell; Treg, regulatory T cell; XIAP, X-linked inhibitor of apoptosis. Bold text in the first column indicates the strategy category.

Recent technological advances, including high-throughput sequencing and spatial imaging, have illuminated the multifaceted interactions between viruses and the TIME at unprecedented resolution. These studies reveal that viruses not only serve as pathogens or therapeutic agents but also act as immune modulators influencing pattern recognition receptor (PRR) signaling, interferon pathways, metabolic reprogramming, and epigenetic regulation within the tumor milieu. For instance, the oncolytic swine pseudorabies virus attenuated vaccine (PRV-LAV) has been shown to infect tumor cells via the NRP1/EGFR pathway, induce endoplasmic reticulum stress-mediated apoptosis, and reprogram the TIME by enhancing immune cell infiltration and restoring CD8+ T cell function. When combined with ICIs, PRV-LAV exhibits synergistic antitumor effects, highlighting the intricate crosstalk between viral infection and immune modulation in the TIME (48). Similarly, engineering strategies to improve systemic delivery of OVs, such as incorporating CD47 extracellular domains to evade mononuclear phagocyte system clearance and enhancing resistance to neutralizing antibodies, are advancing the clinical applicability of virotherapy beyond localized intratumoral injections (46).

While T-VEC has been approved for melanoma and demonstrated efficacy both as monotherapy and in combination with ICIs, response rates vary, and resistance mechanisms persist. Tumor heterogeneity, immune suppression, antiviral immunity, and delivery barriers limit the full therapeutic potential of OVs. Ongoing research focuses on next-generation viral constructs with enhanced tumor selectivity, immune-stimulatory payloads, and combinatorial regimens integrating antiviral and antitumor immunity. For example, combining OVs with small molecule inhibitors or anti-angiogenic agents has shown promise in preclinical and clinical settings, aiming to potentiate immune activation and overcome immunosuppressive networks within the TIME (1, 8). Moreover, the integration of artificial intelligence and biomarker-driven approaches is anticipated to personalize OV-based therapies, optimizing patient selection and therapeutic efficacy (2).

This review is organized to follow the two directions of virus-TIME interaction in turn and then their convergence. Section 2 examines how viruses remodel the cellular composition of the TIME, covering tumor-infiltrating lymphocytes, tumor-associated macrophages and myeloid-derived suppressor cells. Section 3 addresses cytokine networks, separating the proinflammatory circuits engaged by PRR signaling and type I interferon from the immunosuppressive circuits sustained by persistent infection. Section 4 covers the immune evasion programs of oncogenic viruses, namely interference with MHC-I presentation, suppression of NK cell activating signals and induction of inhibitory checkpoints, which are summarized in **Table 2**. Sections 5–8 turn to therapeutic exploitation: viral antigens as immunotherapeutic targets and viral vector vaccine design (Section 5), OV-induced ICD and disruption of immunosuppressive networks (Section 6), viral vector delivery of checkpoint inhibitors, cytokines and costimulatory ligands (Section 7), and combination regimens with PD-1/PD-L1 or CTLA-4 blockade (Section 8), with the therapeutic strategies consolidated in **Table 1**. Section 9 considers the metabolic and epigenetic layer that underlies both directions, and Section 10 turns to clinical translation, treating mechanisms of resistance first, since they define what a biomarker has to measure, and then biomarker development and safety management. The concluding section integrates the two virus categories, setting out where their effects on the TIME oppose one another and where they can be combined deliberately.

**Table 2:** Mechanisms by which oncogenic viruses reshape the tumor immune microenvironment for immune evasion.

Virus	Associated malignancy	Key viral effector(s)	Hijacked host pathway	Immune cells affected	Upregulated immunosuppressive molecules	Downregulated/inactivated molecules	Net immune-evasion outcome	Therapeutic targets/strategies
**HPV** Human papillomavirus *(high-risk: HPV16/18)*	• Cervical carcinoma • Head-and-neck squamous cell carcinoma (HNSCC) • Anal carcinoma • Vulvar neoplasia	**Oncoproteins:** • E5, E6, E7 **Host effector co-opted:** • MARCHF8 (E3 ubiquitin ligase, upregulated by HPV)	• MARCHF8-mediated ubiquitination and lysosomal degradation of MHC-I (53) • Selective autophagy via NDP52/ULK1/PIK3C3 (53) • E7 suppresses cGAS–STING (pRB-binding domain) (54) • E6/E7 transcriptionally upregulate PD-L1 on MDSCs (55, 56) • STAT3, PI3K-AKT-mTOR, Wnt signaling drive MDSC recruitment (57)	• CD8^+^ T cells (impaired recognition) • MDSCs (expanded; enhanced suppressive function) • Tregs (increased infiltration) • Dendritic cells (impaired Ag presentation)	• PD-L1 (E5/E6/E7 cooperative upregulation) • MARCHF8 • ARG1, iNOS, ROS (MDSC-derived)	• Surface MHC-I expression • cGAS–STING signaling • Antigen-processing machinery (TAP, β2m)	• Loss of CD8^+^ T-cell tumor recognition • ICI resistance despite PD-L1 positivity (due to MHC-I loss) (58, 59) • Sustained immunosuppressive TIME enabling viral persistence and progression	• MARCHF8 ablation + anti–PD-1 (overcomes ICI refractoriness) (53) • E7-targeted TCR-T therapy • Multi-epitope (E5/E6/E7) VLP vaccines • Therapeutic DNA vaccine + gemcitabine (MDSC depletion) (60)
**EBV** Epstein–Barr virus *(γ-herpesvirus, HHV-4)*	• Nasopharyngeal carcinoma (NPC) • EBV-associated gastric carcinoma (EBVaGC) • Hodgkin and non-Hodgkin lymphomas • Burkitt lymphoma	**Latency proteins:** • LMP1, LMP2, EBNA2 **Viral miRNAs** **Splicing factor:** • FAM50A (host, hijacked) (61)	• LMP1 → JAK/STAT3 → autocrine IL-10 loop (62, 63) • EBNA2 recruits EZH2/HDACs to remodel chromatin • FAM50A-driven RNA-splicing reprogramming sustains oncogenic STAT3 (61) • Aberrant DNA methylation silences IFN-γ pathway (EBVaGC) (51)	• Macrophages (M2 skewing) • CD8^+^ T cells (impaired recognition; exhaustion) • Dendritic cells • MDSCs	• IL-10 (LMP1/STAT3-driven) • TGF-β • MMP9 (M2-derived) (64) • PD-L1	• MHC-I expression (HDAC-mediated) • IFN-γ signaling (silenced by DNA methylation in EBVaGC) • Antigen presentation	• M2-dominated immunosuppressive TIME • Resistance to TCR-T therapy (64) • Persistent infection and lymphomagenesis • Failure of anti-tumor immunosurveillance	• JAK/STAT3 inhibitors • Adenovirus-LMP2 vaccine (Phase I validated) (34) • HDAC inhibitors + oncolytic virus combinations (49, 50) • LMP1/LMP2-directed TCR-T • Hypomethylating agents (decitabine)
**HBV** Hepatitis B virus *(hepadnavirus)*	• Hepatocellular carcinoma (HCC) • HCC arising on a background of chronic HBV-driven inflammation	**Oncoprotein:** • HBx (central driver) **Viral antigens:** • HBsAg • Chronic persistent antigenic stimulation	• HBx → PI3K/AKT/mTOR → transcriptional PD-L1 upregulation (65) • HIF-1α–driven Warburg effect (aerobic glycolysis) (66) • Mitochondrial ROS accumulation → PD-1↑, IFN-γ↓ • Eomes-driven transcriptional program enforcing T-cell exhaustion	• CD8^+^ T cells (profound exhaustion) • NK cells (exhausted; TIGIT/TIM-3 co-expression) • Tregs (expanded) • MDSCs • Intrahepatic TRM cells (modulated)	• PD-L1 (HBx-driven) • PD-1, TIM-3, LAG-3 co-expression on T cells • Galectin-9 (NK-derived, drives TIM-3 on T cells) • Lactate (Warburg metabolite)	• T-cell IFN-γ production • Proliferative capacity and cytotoxicity • Mitochondrial fitness	• Dual T-cell and NK-cell exhaustion • Refractoriness to anti–PD-1/PD-L1 monotherapy • Acidified, metabolically hostile TIME further suppresses CTL function • Viral persistence and HCC progression	• Combinatorial PD-1 + TIM-3 + LAG-3 blockade • Anti–PD-1 (camrelizumab) + oncolytic virus (44) • Metabolic modulation (ROS scavenging, mitochondrial rescue) • PI3K/AKT/mTOR inhibition (e.g., PRXL2B silencing synergizes with OAd H101) (65)
**HCMV** Human cytomegalovirus *(β-herpesvirus, HHV-5)*	• Glioblastoma (association documented) • Modulator of NK-cell surveillance across multiple solid tumors	**Viral protein:** • UL16 (67) • Additional NK-ligand modulators	• UL16 intracellularly sequesters NKG2D ligands (ULBPs), blocking surface delivery (67) • Shedding of soluble NKG2D ligands (MICA/B, ULBPs) as decoys → NKG2D receptor downregulation (68, 70, 71)	• NK cells (primary target) • NKG2D^+^ CD8^+^ T cells (collateral effect)	• Soluble ULBPs/MICA/B (decoy ligands) • HLA-E, HLA-G (non-classical MHC-I engaging NK inhibitory receptors) (68)	• Surface NKG2D ligand display • NK-cell NKG2D receptor function • NK cytotoxicity and cytokine output	• Loss of NK-mediated immunosurveillance • Escape of virus-infected and transformed cells from NK lysis • Correlation with poor prognosis in glioblastoma, HCC, CRC (67, 70, 71)	• Anti–soluble-ULBP6 antibody (e.g., 23ME-01473) (69) • FcγRIIIa-engaging NK activators • Decitabine to restore NKG2D ligand expression (IDH-mutant glioma) (52) • Allogeneic NK-cell adoptive transfer (72)
**KSHV/HHV-8** Kaposi-sarcoma–associated herpesvirus *(γ-herpesvirus)*	• Kaposi sarcoma • Primary effusion lymphoma • Multicentric Castleman disease	**Splicing reprogrammer:** • FAM50A (host, hijacked) (61) **Viral miRNAs** **Latent gene products**	• Reprogramming of host RNA splicing to sustain oncogenic STAT3 (61) • Proposed STAT3-dependent maintenance of M2 macrophage phenotype • miRNA-mediated metabolic and signaling rewiring	• Macrophages (sustained M2 polarization) • T cells (impaired effector function)	• IL-10, TGF-β (M2-derived) • Persistently activated STAT3 signaling	• M1-polarization signals • Anti-tumor cytokines	• TAM-dominated immunosuppressive TIME • Support for tumor outgrowth and viral latency maintenance	• STAT3 inhibitors • FAM50A-directed splicing-modulator strategies • CSF1R inhibitors to deplete M2 TAMs
**HCV** Hepatitis C virus *(Flaviviridae)*	• Hepatocellular carcinoma • Chronic HCV-associated liver disease	• Chronic inflammatory cytokine milieu **Host lncRNA upregulated by HCV:** • HOTAIRM1 (73)	• HOTAIRM1 → HOXA1–miR-124 axis drives MDSC differentiation/expansion (73) • Upregulation of STAT3, iNOS, ARG1 in MDSCs • Chronic inflammation–driven bone-marrow mobilization of MDSCs	• MDSCs (massively expanded; enhanced suppressive function) • CD8^+^ T cells (functional suppression) • NK cells	• ARG1, iNOS, ROS (MDSC-mediated) • PD-L1 (MDSC surface) • STAT3	• L-arginine (depleted by ARG1) • TCR signaling • T-cell proliferative capacity	• Canonical MDSC-mediated T-cell suppression • Sustained viral infection contributing to hepatocarcinogenesis • Constrained responsiveness to immunotherapy	• HOTAIRM1/HOXA1-axis targeting • MDSC-depleting agents (gemcitabine) • PI3Kγ inhibitors (74) • Combination with anti–PD-1 (43)
**HIV** Human immunodeficiency virus *(Retroviridae, lentivirus)*	• AIDS-related non-Hodgkin lymphoma • AIDS-related Kaposi sarcoma • Elevated incidence of EBV/HPV/KSHV-associated malignancies	• Chronic viral antigen stimulation • Systemic immune exhaustion and dysregulated cytokine networks	• Persistent antigen-driven T-cell exhaustion • Sustained upregulation of immunosuppressive cytokines • Altered cytokine network homeostasis (66)	• CD4^+^ T cells (depletion) • CD8^+^ T cells (exhaustion) • Dendritic cells (functional impairment)	• TGF-β • IL-10 • VEGF (angiogenesis + recruitment of suppressive cells)	• DC maturation • Antigen presentation • Effector T-cell function	• Systemic failure of immunosurveillance • Increased incidence of co-infection-driven (EBV/HPV/KSHV) malignancies • Therapeutically challenging tumor comorbidities	• Combination antiretroviral therapy + immuno-oncology • Combined TGF-β/IL-10/VEGF pathway blockade • Treatment of co-infecting oncogenic viruses

Ag, antigen; ARG1, arginase-1; CRC, colorectal cancer; CTL, cytotoxic T lymphocyte; DC, dendritic cell; EBVaGC, EBV-associated gastric carcinoma; HCC, hepatocellular carcinoma; HDAC, histone deacetylase; HNSCC, head-and-neck squamous cell carcinoma; ICI, immune checkpoint inhibitor; IDH, isocitrate dehydrogenase; IFN, interferon; LMP, latent membrane protein; MDSC, myeloid-derived suppressor cell; MPS, mononuclear phagocyte system; NK, natural killer; NPC, nasopharyngeal carcinoma; OAd, oncolytic adenovirus; PRR, pattern-recognition receptor; ROS, reactive oxygen species; TAP, transporter associated with antigen processing; TCR-T, T-cell receptor–engineered T cell; TIME, tumor immune microenvironment; TRM, tissue-resident memory T cell; ULBP, UL16-binding protein; VLP, virus-like particle. Bold text indicates the virus (first column) and the class of viral effector within a cell.

## 2 Remodeling of tumor immune microenvironment cell composition by viruses

### 2.1 Recruitment and functional regulation of tumor-infiltrating lymphocytes

Virus infections significantly influence the recruitment and functional modulation of tumor-infiltrating lymphocytes (TILs), particularly CD8+ T cells and natural killer (NK) cells, within the tumor immune microenvironment. Viral infection of tumor cells can induce the secretion of chemokines such as CXCL9, CXCL10, and CCL5, which are critical for attracting effector immune cells to the tumor site. These chemokines establish gradients that promote the infiltration of CD8+ cytotoxic T lymphocytes (CTLs) and NK cells, both essential for antitumor immunity. For example, in HBV-related hepatocellular carcinoma (HCC), the viral presence stimulates chemokine release, facilitating the recruitment of these immune effectors into the tumor microenvironment, thereby potentially enhancing immune surveillance and tumor control (75, 76).

However, chronic viral infections, such as persistent HBV infection, often lead to a state of T cell exhaustion characterized by impaired effector functions and sustained expression of multiple inhibitory receptors including programmed cell death protein 1 (PD-1), T cell immunoglobulin and mucin domain-containing protein 3 (TIM-3), and lymphocyte activation gene 3 (LAG-3) (77, 78). The transcription factor Eomesodermin (Eomes) has been identified as a key regulator promoting the expression of these inhibitory receptors, thereby reinforcing the exhausted state of hepatic CD8+ T cells during chronic HBV infection (79, 80). Moreover, the co-expression of PD-1, TIM-3, and LAG-3 on HBV-specific CD8+ T cells correlates with functional impairment and resistance to monotherapy with PD-1/PD-L1 blockade, suggesting that combinatorial immune checkpoint inhibition may be necessary to restore effective antiviral and antitumor immunity (81–83).

In addition to T cells, NK cells also play a pivotal role in the antiviral and antitumor immune response within the HBV-infected liver (84). However, in chronic HBV infection and HBV-related HCC, NK cells often exhibit an exhausted phenotype marked by co-expression of inhibitory receptors such as TIGIT and TIM-3, leading to reduced cytotoxicity and cytokine secretion. The interaction between NK cells and CD8+ T cells is complex; for instance, NK cells expressing high levels of galectin-9 (Gal-9) can engage TIM-3 on CD8+ T cells, further driving T cell dysfunction and exhaustion (85).

Therapeutic viruses act on this exhaustion program in both directions, and the direction is platform-dependent. Oncolytic adenoviruses and oncolytic herpesviruses engage innate sensing pathways, notably the stimulator of interferon genes (STING) pathway, and the resulting type I interferon response can partially restore effector function in exhausted T cells, providing a rationale for combining viral oncolysis with immune checkpoint blockade in virus-associated tumors (86, 87). Oncolytic viruses are not uniformly reinvigorating, however. Oncolytic vaccinia virus has been shown to inactivate intratumoral dendritic cells and to drive a TIM-3-high exhausted T cell phenotype, with antitumor efficacy recovered only when TIM-3 is blocked (88). The exhaustion mechanisms described in this section therefore refer to chronic infection by oncogenic viruses as the driver, whereas therapeutic viruses may relieve or aggravate exhaustion depending on the platform, the dosing schedule and the pre-existing immune context; this distinction is maintained throughout the sections that follow.

Furthermore, metabolic reprogramming within the tumor microenvironment, influenced by viral infection, also affects TIL function. Chronic HBV infection induces mitochondrial reactive oxygen species (ROS) accumulation in T cells, which correlates with increased PD-1 expression and diminished interferon-gamma (IFN-γ) production, exacerbating T cell exhaustion. Targeting metabolic pathways to reduce oxidative stress and restore mitochondrial function may therefore represent an adjunctive strategy to enhance TIL efficacy (89). Such metabolic pressure on T cells need not originate from the virus itself. In esophageal squamous cell carcinoma, indole-3-lactic acid produced by the gut commensal *Ligilactobacillus salivarius* activates the aryl hydrocarbon receptor and suppresses NF-κB signaling in NKG7+CD8+ precursor-exhausted T cells, driving terminal exhaustion and resistance to anti-PD-1 therapy (90).

In summary, persistent infection by oncogenic viruses shapes TIL recruitment and function through chemokine-mediated trafficking, sustained inhibitory receptor expression, metabolic stress and NK–T cell crosstalk, whereas therapeutic viruses can partially reverse, or for some platforms deepen, the same dysfunction. Strategies under investigation include combinatorial checkpoint blockade, metabolic modulation and oncolytic virotherapy, and their rational design depends on specifying which of these two very different viral contexts is being targeted.

### 2.2 Tumor-associated macrophages polarization reprogramming

Tumor-associated macrophages (TAMs) are a critical component of the TIME, exhibiting remarkable plasticity that allows them to polarize into distinct functional phenotypes, primarily the pro-inflammatory M1 type and the immunosuppressive M2 type. Viral components, such as double-stranded RNA (dsRNA) and viral proteins, are recognized by pattern recognition receptors (PRRs) on macrophages, including Toll-like receptor 3 (TLR3) and retinoic acid-inducible gene I (RIG-I). This recognition triggers signaling cascades that promote M1 polarization, characterized by the secretion of pro-inflammatory cytokines such as tumor necrosis factor-alpha (TNF-α) and interleukin-12 (IL-12), which exert potent antitumor effects. This M1 polarization is crucial for initiating effective antitumor immune responses and enhancing the efficacy of immunotherapies. In murine Lewis lung carcinoma and B16 melanoma models, administration of the TLR3 agonist poly-ICLC promoted M1 polarization of TAMs and restrained tumor growth, providing direct *in vivo* support for this axis (91).

Conversely, certain oncogenic viruses, notably EBV, have evolved sophisticated mechanisms to subvert macrophage polarization towards the M2 phenotype, which supports tumor progression by suppressing antitumor immunity. EBV encodes viral microRNAs (miRNAs) and latent proteins that manipulate host cellular pathways to promote M2 polarization of TAMs. For example, EBV latent membrane proteins can induce autocrine IL-10 production in macrophages, reinforcing the M2 phenotype and creating an immunosuppressive microenvironment that impairs T cell function and contributes to resistance against T cell receptor-engineered T cell (TCR-T) therapies. The secretion of matrix metalloproteinase 9 (MMP9) by EBV-induced M2 macrophages further exacerbates immune suppression and tumor invasiveness, highlighting the complex interplay between viral factors and TAM polarization in tumor immune escape (64).

Two classes of TAM-directed intervention are relevant here, and they differ in whether the agent is itself a virus. The first is the viral PRR agonism described above, which favors M1 repolarization of TAMs during oncolysis and contributes to the antitumor activity of several OV platforms (9). Second, non-viral agents acting directly on macrophage phenotype have been developed in parallel and are summarized here as mechanistic comparators rather than as virus-based therapies: exosomes derived from M1 macrophages repolarize M2 TAMs and restore phagocytic and proinflammatory function (92), and bacterial adhesion proteins such as FimH drive M1 polarization through TLR4 signaling and reverse M2-associated immunosuppression, thereby enhancing responses to immune checkpoint inhibitors (93).

At the molecular level, oncogenic γ-herpesviruses act both on the host machinery that sustains oncogenic signaling and on macrophages themselves. KSHV reprograms host RNA splicing through the splicing factor FAM50A to sustain STAT3 activation and drive oncogenic cellular transformation (61). Independently of that mechanism, KSHV is an established modulator of monocyte and macrophage behavior in the tumor microenvironment (94), and KSHV-encoded microRNAs induce IL-6 and IL-10 secretion by human monocytes and macrophages, an output consistent with M2-like skewing (55). Taken together, these findings indicate that γ-herpesviruses influence TAM phenotype through both virus-encoded non-coding RNAs and latent proteins; direct evidence that FAM50A-dependent splicing itself controls macrophage polarization is currently lacking, and the two mechanisms are therefore presented separately rather than as a single pathway.

### 2.3 Expansion and functional suppression of myeloid-derived suppressor cells

Myeloid-derived suppressor cells (MDSCs) represent a heterogeneous population of immature myeloid cells that expand significantly under pathological conditions, including chronic viral infections and cancer, where they exert potent immunosuppressive functions. Viral infections can induce chronic inflammation, which acts as a critical driver for the mobilization and expansion of MDSCs from the bone marrow. Mechanistically, MDSCs suppress T cell function through multiple pathways, including the production of ROS, arginase-1 (ARG1), and nitric oxide (NO). These molecules interfere with T cell receptor signaling, deplete essential amino acids such as L-arginine, and induce T cell apoptosis or anergy, thereby dampening effective antiviral and antitumor immune responses (56, 95). In the context of viral oncogenesis, such as HPV-associated head and neck squamous cell carcinoma (HNSCC), viral oncoproteins E6 and E7 have been shown to directly upregulate programmed death-ligand 1 (PD-L1) expression on MDSCs, enhancing their immunosuppressive capacity. This upregulation of PD-L1 facilitates immune evasion by engaging PD-1 on T cells, leading to T cell exhaustion and impaired cytotoxic activity (57, 96).

OV therapy, which employs replication-competent viruses to selectively infect and lyse tumor cells, has emerged as a promising immunotherapeutic approach. OVs can modulate the tumor microenvironment by inducing immunogenic cell death and releasing damage-associated molecular patterns (DAMPs), which activate dendritic cells and promote antitumor immunity. Importantly, OV-induced tumor cell apoptosis can partially reverse the suppressive activity of MDSCs by altering their phenotype and function. For example, oncolytic vaccinia virus (OVV) infection combined with embelin, an inhibitor of XIAP, was shown to enhance viral replication and reduce MDSC infiltration in lymphoma models, thereby improving antitumor immune responses and survival (24). Similarly, IL-36γ-armed oncolytic vaccinia viruses decreased MDSC and M2-like macrophage populations while promoting effector T cell differentiation, resulting in potent adaptive antitumor immunity (23). However, the extent to which OV therapy can reverse MDSC-mediated immunosuppression varies depending on the virus type and the tumor context. In GM-CSF-cultured murine bone marrow-derived dendritic cells, a heat-inactivated oncolytic vaccinia virus induced IFN-β together with the chemokines CCL5 and CXCL10 in a STING-dependent manner, whereas the live virus induced neither IFN-β nor CXCL10 and raised CCL5 only marginally; *in vivo* this corresponded to more effective systemic antitumor immunity and a greater reduction in MDSCs (97). The interaction is reciprocal and sensor-dependent rather than a uniform property of OVs: intratumoral MDSCs deplete arginine and thereby inhibit OV replication (98), whereas oncolytic reovirus suppresses MDSC-mediated inhibition strictly through TLR3 (99). These findings suggest that the immunomodulatory effects of OVs on MDSCs are complex and influenced by viral replication competence and the nature of the immune activation elicited.

In HPV-associated malignancies, therapeutic strategies combining immunotherapy with agents targeting MDSCs have demonstrated synergistic effects. For instance, the combination of a DNA vaccine targeting HPV-16 E7 oncoprotein with gemcitabine, a chemotherapeutic known to reduce MDSC accumulation, resulted in enhanced antigen-specific CD8+ T cell responses and tumor eradication in preclinical models. This combination also decreased the infiltration of immunosuppressive MDSCs and regulatory T cells within the tumor microenvironment, highlighting the importance of modulating MDSC populations to overcome immune suppression (60).

MDSC expansion is a general feature of chronic viral infection as well as of cancer, and the effector pathways involved, including ARG1, inducible nitric oxide synthase (iNOS), STAT3 and ROS, overlap with those operating in virus-associated tumors (73, 100). Because the present review is concerned with the tumor immune microenvironment, the literature on MDSCs in non-oncogenic infection is not surveyed further.

The suppressive program outlined above is itself subject to epigenetic control: DNA methylation at the promoters of genes encoding ARG1, TGF-β, TIM-3 and related immunosuppressive molecules modulates MDSC differentiation and function (101). MDSCs are also plastic, adapting to the inflammatory and metabolic conditions of the tumor and differentiating into other myeloid cells with distinct roles, which complicates therapeutic targeting (102).

The plasticity that complicates MDSC targeting also creates an opening for agents that treat MDSCs as antigen-bearing cells rather than as a functional state to be reversed. B7-H4 is of interest in this respect because it is expressed not only on tumor cells but also on suppressive tumor-associated macrophages and on MDSCs, where its induction is driven by the same IL-6, IL-10 and STAT3 signals that oncolytic infection amplifies (103, 104). Three B7-H4-directed antibody-drug conjugates, XMT-1660, AZD8205 and SGN-B7H4V, have entered phase I evaluation in solid tumors (103). Because an oncolytic virus that lowers MDSC number or suppressive activity (23, 99) still leaves a residual, and in some settings virus-recruited (105, 106), myeloid compartment, coupling oncolysis to cytotoxic delivery against a marker enriched on that compartment is a rational extension: the virus supplies the inflammatory signal that upregulates the target, and the conjugate removes the cells that would otherwise blunt the response. No such combination has been reported, and two specific risks would have to be resolved experimentally before it could be recommended. B7-H4 is also expressed on antigen-presenting cells whose function the virus depends on, and the payloads carried by these conjugates are antimitotic, so their effect on an expanding virus-specific and tumor-specific T cell pool cannot be predicted from tumor cell data.

Emerging therapeutic approaches aim to target MDSCs to enhance antitumor immunity and improve responses to immunotherapy. Strategies include depletion of MDSCs, inhibition of their recruitment and expansion, blockade of their suppressive functions, and promotion of their differentiation into mature myeloid cells. For example, PI3Kγ inhibitors reduce MDSC-mediated immunosuppression and synergize with photodynamic therapy to enhance CD8+ T cell infiltration and tumor control (74). Oncolytic virotherapy combined with agents that modulate MDSC activity holds promise for overcoming resistance to immune checkpoint inhibitors (43), although the net effect of an OV on the MDSC compartment is platform- and tumor-dependent: some constructs reduce MDSC numbers or suppressive activity (23, 99), whereas others recruit MDSCs through virus-induced IL-6 and IL-8 (105, 106). Additionally, adoptive transfer of *in vitro*-generated MDSCs with controlled suppressive activity is being explored for immune regulation in transplantation and autoimmune diseases, highlighting the dual role of MDSCs in immune homeostasis and pathology (107) (**Figure 1**).

**Figure 1:**
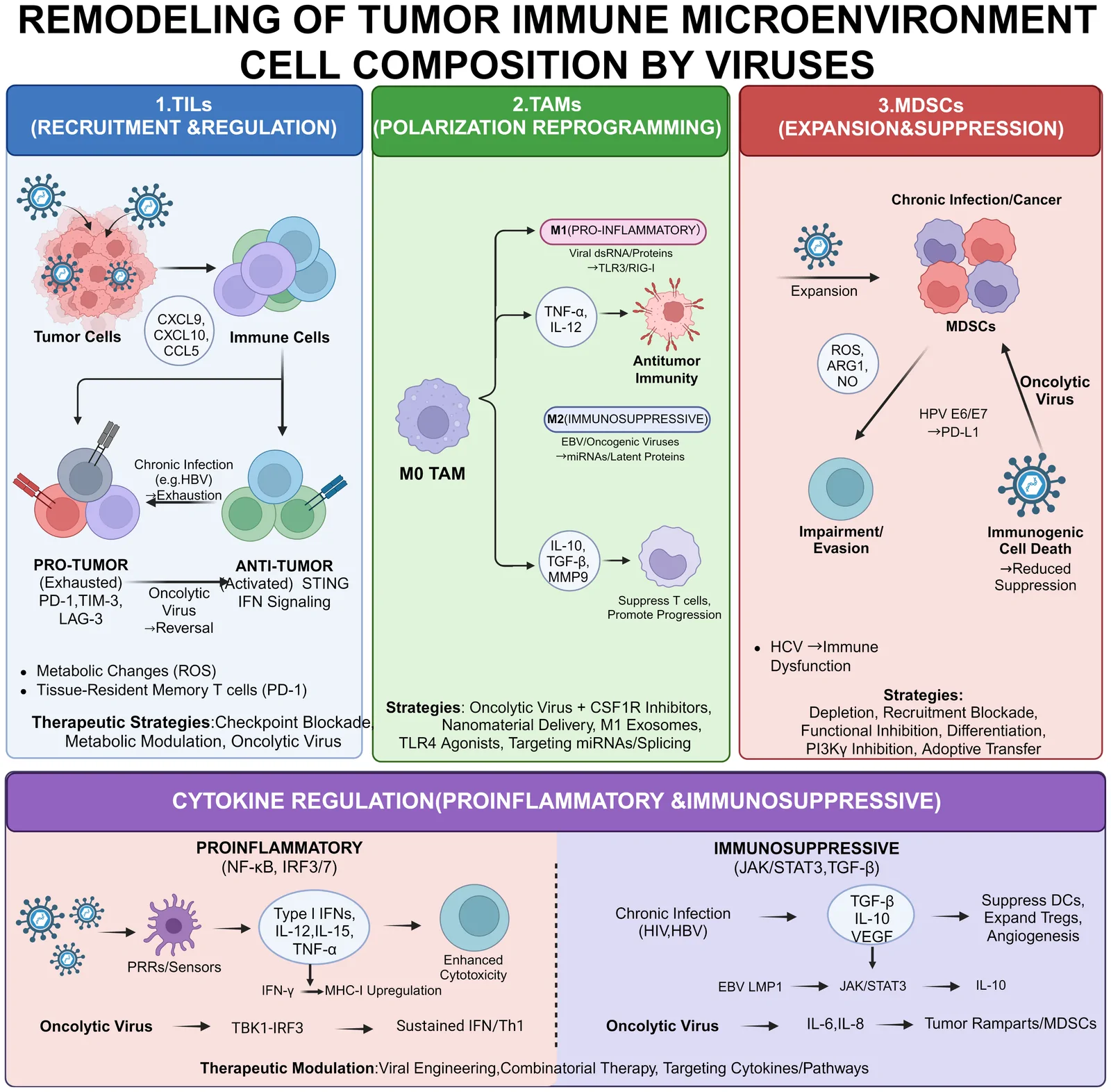
Remodeling of tumor immune microenvironment by viruses. Illustrates the modulation of TILs, TAMs, and MDSCs by viral infection. Viruses recruit and activate CD8+ T cells and NK cells via chemokine signaling (e.g., CXCL9, CXCL10, CCL5), polarize TAMs towards pro-inflammatory M1 or immunosuppressive M2 phenotypes, and regulate MDSC expansion and suppressive function. Therapeutic strategies including oncolytic virus therapy, checkpoint blockade, and metabolic modulation are depicted to highlight their effects on restoring antitumor immunity and reshaping the tumor immune microenvironment. Throughout the figure, mechanisms attributable to persistent infection by oncogenic viruses (HBV, EBV, HPV, KSHV) are distinguished from those attributable to therapeutic oncolytic viruses; unless indicated otherwise in the corresponding text, the underlying evidence derives from murine models.

## 3 Regulation of tumor immune microenvironment cytokines by viruses

### 3.1 Induction of proinflammatory cytokines and activation of antitumor immunity

Viral infections within the TIME initiate a cascade of innate immune signaling pathways that culminate in the production of proinflammatory cytokines, which are pivotal for activating antitumor immune responses. Central to this process is the activation of transcription factors such as nuclear factor kappa-light-chain-enhancer of activated B cells (NF-κB) and interferon regulatory factors 3 and 7 (IRF3/7). Upon viral recognition by PRRs including Toll-like receptors (TLRs) and cytosolic sensors like RIG-I and cGAS-STING, these pathways are engaged, leading to the robust induction of type I interferons (IFN-α/β) and other key cytokines such as IL-12, interleukin-15 (IL-15), and TNF-α. For instance, studies have demonstrated that OVs, such as recombinant poliovirus and myxoma virus, stimulate TBK1-IRF3 signaling pathways, resulting in sustained type I/III interferon release that primes polyfunctional, Th1-skewed antitumor T cell responses, distinct from NF-κB-polarized TLR activation (9).

The role of type I interferons extends beyond direct antiviral effects; they act as potent immunomodulators that enhance antigen presentation and T cell priming, and their induction is a shared requirement of the OV platforms discussed in Sections 6–8 (9). Conversely, many viruses encode proteins that interrupt this axis at the level of IRF3 phosphorylation. The balance between interferon induction and viral interferon antagonism is thus a principal determinant of both oncogenic persistence and oncolytic efficacy.

Oncolytic virotherapy leverages these innate immune activations to remodel the TIME. For example, infection with oncolytic herpes simplex virus (HSV) or vaccinia virus can induce phosphorylation of IRF3, leading to enhanced expression of interferon-stimulated genes (ISGs) and improved tumor antigen presentation. Genetic modifications of OVs to remove immune evasion genes have been shown to rescue IRF3 activation, thereby augmenting cytotoxic T lymphocyte (CTL) responses against tumor-associated antigens and improving antitumor efficacy *in vivo* (108). Moreover, combinatorial approaches using OVs with ICIs or JAK inhibitors can modulate the antiviral type I IFN response to optimize antitumor immunity without compromising antigen presentation (109).

A notable example of cytokine induction following viral infection in the TIME is the elevation of IFN-γ levels after infection with oncolytic reovirus. IFN-γ plays a crucial role in upregulating major histocompatibility complex class I (MHC-I) molecules on tumor cells, thereby enhancing tumor antigen presentation and facilitating recognition by CD8+ T cells. This phenomenon has been observed in murine models where oncolytic virus infection leads to increased IFN-γ production within the TIME, promoting a more immunogenic milieu conducive to effective antitumor responses (10).

However, the induction of proinflammatory cytokines must be tightly regulated to avoid deleterious effects. Excessive release of cytokines such as IL-6 and IL-1β can produce systemic inflammation and tissue damage, and cytokine-mediated toxicity is a recognized clinical concern for intratumoral virotherapy as well as for severe natural infection. In the context of oncolytic virotherapy, mitigation strategies include engineering viruses to attenuate excessive inflammatory signaling and combining virotherapy with agents that limit cytokine-mediated toxicity (110).

### 3.2 Upregulation of immunosuppressive cytokines and immune escape

Chronic viral infections, such as those caused by HIV and HBV, are well-recognized for their capacity to induce a persistent immunosuppressive milieu within the TIME, primarily through the sustained secretion of immunosuppressive cytokines including TGF-β, IL-10, and vascular endothelial growth factor (VEGF). This cytokine milieu fosters immune escape by tumors, enabling viral persistence and tumor progression in the context of chronic viral infections (4). In HBV-related HCC this is accompanied by intratumoral accumulation of stem-like CCR4+ regulatory T cells that organize the suppressive microenvironment (62).

In EBV-associated malignancies, the viral latent membrane protein 1 (LMP1) is a principal driver of the immunosuppressive landscape. LMP1 constitutively activates NF-κB, PI3K and JAK/STAT signaling. LMP1-dependent PI3K activation induces IL-10 production (63), while LMP1-driven STAT3 activation contributes to the leukocyte-rich yet immunosuppressive microenvironment characteristic of nasopharyngeal carcinoma (111). IL-10 in this setting promotes immune tolerance, restrains effective antitumor responses and supports tumor cell survival and proliferation. This mechanism highlights the interplay between viral oncogenes and host immune regulation and identifies the LMP1-PI3K/STAT3-IL-10 axis as a potential therapeutic target for disrupting the immunosuppressive niche in EBV-related malignancies.

OV therapies, while promising as cancer treatments due to their ability to selectively infect and lyse tumor cells and stimulate antitumor immunity, can paradoxically induce immunosuppressive cytokine production that undermines their efficacy. Tumor cells infected by OVs often release pro-inflammatory cytokines such as IL-6 and interleukin-8 (IL-8). IL-8, in particular, has been implicated in the formation of a spatially organized tumor self-rampart (TSR), a concentric barrier of senescent and fibrotic tumor cells that confines viral spread and limits OV therapeutic effectiveness. Moreover, IL-6 and IL-8 contribute to the recruitment and expansion of MDSCs, which are potent suppressors of antitumor immunity. For example, blockade of IL-8 with Reparixin or the use of glucocorticoids has been shown to disrupt TSR formation, prolong viral persistence, and potentiate OV efficacy in glioblastoma models (105).

## 4 Virus-induced immune evasion mechanisms and their impact on TIME

The principal mechanisms by which the major oncogenic viruses—HPV, EBV, HBV, HCMV, KSHV, HCV, and HIV—reshape the TIME to achieve immune evasion are summarized in **Table 2**.

### 4.1 Viral protein interference with MHC class I molecule presentation

Viruses have evolved sophisticated mechanisms to evade host immune surveillance, particularly by targeting the MHC-I antigen presentation pathway, which is critical for CD8+ CTL recognition and elimination of infected or transformed cells. Several DNA viruses, including adenoviruses and herpesviruses, encode proteins that interfere with the transport and surface expression of MHC-I molecules. The adenovirus E3-19K glycoprotein binds MHC-I molecules and retains them in the endoplasmic reticulum, a mechanism defined structurally by the crystal structure of E3-19K bound to HLA-A2 (58). The herpes simplex virus immediate-early protein ICP47 instead blocks the transporter associated with antigen processing (TAP) and thereby prevents peptide loading onto MHC-I; notably, ICP47 inhibits human but not murine TAP, a species restriction that constrains the inferences that can be drawn from mouse models of this particular mechanism (59). These strategies permit persistent infection and, for the oncogenic viruses considered here, allow transformed cells to escape immune-mediated clearance.

HPV E5 has been demonstrated to downregulate MHC-I surface expression on infected cells, thereby impairing antigen presentation to CD8+ T cells. Concurrently, E5 upregulates the expression of PD-L1, a key immune checkpoint molecule that inhibits T cell activation and promotes immune tolerance. The suppression of MHC-I not only diminishes the effectiveness of cytotoxic T cell responses but also correlates with reduced responsiveness to ICIs, which rely on intact antigen presentation machinery to elicit anti-tumor immunity (112).

Recent studies have elucidated the molecular underpinnings of MHC-I downregulation in HPV-positive malignancies. For example, the membrane-associated RING-CH-type finger 8 (MARCHF8) ubiquitin ligase, upregulated by HPV, ubiquitinates MHC-I molecules, targeting them for degradation (113). Knockdown or genetic ablation of MARCHF8 restores MHC-I surface levels, enhances CD8+ T cell infiltration, and suppresses tumor growth in HPV-positive head and neck cancers (113). Moreover, MARCHF8 knockout synergizes with anti-PD-1 therapy to overcome resistance in immune checkpoint inhibitor-refractory tumors, highlighting the therapeutic potential of targeting this pathway (113).

In cervical and vulvar neoplasms, a significant proportion of tumors exhibit clonal or complete loss of MHC-I expression, including many that are PD-L1 positive. This phenotype suggests that despite the presence of immune checkpoint molecules, the lack of antigen presentation impairs the efficacy of checkpoint blockade therapies (54).

High-risk HPV types, such as HPV16 and HPV18, encode E5 proteins that modulate host gene expression differently from low-risk types, influencing pathways involved in immune regulation and carcinogenesis (53, 114). Variants of the E5 oncogene exhibit polymorphisms that affect protein structure, immunogenic epitopes, and signaling pathways such as NF-κB, which may alter the degree of MHC-I downregulation and immune escape (115). Experimental models targeting E5 to dendritic cells have demonstrated the induction of robust CD4+ and CD8+ T cell responses and tumor protection, suggesting that E5 remains a promising target for therapeutic vaccine development despite its immune modulatory functions (116, 117).

In addition to HPV, other DNA viruses such as adenovirus and herpesviruses employ similar strategies to subvert MHC-I presentation. For instance, adenovirus E1A and HPV E7 proteins share a retinoblastoma protein (pRB) binding domain and inhibit the STING pathway, a critical component of innate immunity. Post-translational modifications like protein S-nitrosylation of these viral proteins can limit their ability to inhibit STING, representing a host defense mechanism that counteracts viral immune evasion (70).

### 4.2 Virus-mediated suppression of NK cell activation signals

Viruses have evolved sophisticated mechanisms to evade immune surveillance, particularly by NK cells, which are critical effectors in the innate immune response against tumors and infected cells. One prominent viral strategy involves the modulation of NK cell activating signals through interference with the expression and function of ligands for activating receptors such as NKG2D and NKp30. Viruses can suppress NK cell activation by upregulating non-classical MHC class I molecules, including HLA-E and HLA-G, or by releasing soluble forms of NKG2D ligands, which act as decoys to inhibit receptor engagement on NK cells. This immune evasion tactic effectively diminishes NK cell cytotoxicity and cytokine production, facilitating viral persistence and contributing to tumor immune escape within the TIME; these mechanisms, of which proteolytic shedding of NKG2D ligands is the best characterized, are considered in turn below (71).

A well-characterized example is human cytomegalovirus (HCMV), which encodes the UL16 protein that binds and sequesters NKG2D ligands such as UL16-binding proteins (ULBPs), preventing their surface expression and subsequent recognition by NK cells. This sequestration impairs NK cell-mediated killing of infected or transformed cells, thereby promoting viral survival and potentially aiding tumor progression in virus-associated malignancies (67).

Beyond direct ligand sequestration, viruses can induce the shedding of soluble NKG2D ligands, such as MICA/B and ULBPs, which bind to NKG2D receptors on NK cells and downregulate their surface expression, leading to NK cell functional exhaustion. Elevated levels of soluble NKG2D ligands have been correlated with poor clinical outcomes in various cancers, including glioblastoma and colorectal cancer, where tumor cells exploit this mechanism to evade NK cell surveillance (68, 118).

In the TIME, virus-induced suppression of NK cell function provides an additional layer of immune escape for tumors. For example, in HCC, downregulation and intracellular retention of NKG2D ligands such as ULBP3 have been observed under hypoxic conditions, a state often exacerbated by viral infections like hepatitis viruses. This retention prevents ligand presentation on the tumor cell surface, thereby reducing NK cell activation and facilitating tumor immune evasion (119).

Therapeutic interventions targeting the restoration of NKG2D ligand expression or blocking soluble ligand-mediated suppression have shown promise. Antibodies such as 23ME-01473, which block soluble ULBP6-mediated immunosuppression and enhance NK cell activation via FcγRIIIa engagement, exemplify novel approaches to counteract viral and tumor-induced NK cell inhibition (69).

### 4.3 Virus-induced upregulation of immune checkpoint molecules

Chronic viral infections are well-recognized for their capacity to modulate the tumor immune microenvironment by inducing the upregulation of immune checkpoint molecules such as PD-L1, cytotoxic T-lymphocyte-associated protein 4 (CTLA-4), and LAG-3 on both tumor cells and immune cells. This phenomenon creates an “immune checkpoint barrier” that facilitates immune evasion by tumors, particularly in virus-associated cancers like HCC linked to HBV infection. Persistent viral antigen stimulation during chronic infection leads to T cell exhaustion, characterized by the inhibitory receptor program described in Section 2.1, together with concomitant upregulation of their ligands on tumor and stromal cells, thereby dampening effective antitumor immunity. In HBV-related HCC, the viral X protein (HBx) plays a pivotal role in this immune modulation by directly activating oncogenic signaling pathways such as the phosphoinositide 3-kinase (PI3K)/Akt/mammalian target of rapamycin (mTOR) axis, which transcriptionally enhances PD-L1 expression on tumor cells. This mechanistic link underscores how viral proteins can hijack host signaling to establish an immunosuppressive microenvironment conducive to tumor progression (65).

OV therapies, designed to selectively infect and lyse tumor cells while stimulating antitumor immunity, paradoxically also induce immune checkpoint molecule upregulation.

Further clinical evidence supports the dual role of OVs in modulating immune checkpoints. In advanced HCC patients, combination therapies involving OVs and immune checkpoint inhibitors such as camrelizumab (anti-PD-1) have demonstrated promising safety profiles and efficacy, with high response rates and manageable adverse events. These findings imply that while OVs can induce immune checkpoint molecule expression, their combination with checkpoint blockade can potentiate antitumor immunity by reversing T cell exhaustion and overcoming the immunosuppressive tumor microenvironment (44).

The induction of immune checkpoint molecules by viruses is not limited to PD-L1 and CTLA-4 but extends to other inhibitory receptors such as LAG-3 and CD39, which have been identified as additional therapeutic targets in HCC. Notably, repeated dosing of OVs may exacerbate immune-mediated liver toxicity due to excessive T cell infiltration in the background liver, without enhancing therapeutic efficacy, indicating that careful scheduling and dosing are critical to balance immune activation and checkpoint-mediated suppression (45).

At the molecular level, the PI3K/Akt/mTOR pathway emerges as a central axis linking viral oncogenesis and immune checkpoint regulation. For example, silencing of PRXL2B, a gene upregulated in HCC and associated with poor prognosis, reduces AKT phosphorylation and PD-L1 expression, enhancing the antitumor effect of oncolytic adenovirus H101. Additionally, oncolytic vaccinia viruses armed with immunomodulatory molecules can activate type I interferon responses and modulate antiviral defense pathways, further influencing checkpoint expression and immune cell recruitment (120) (**Figure 2**).

**Figure 2:**
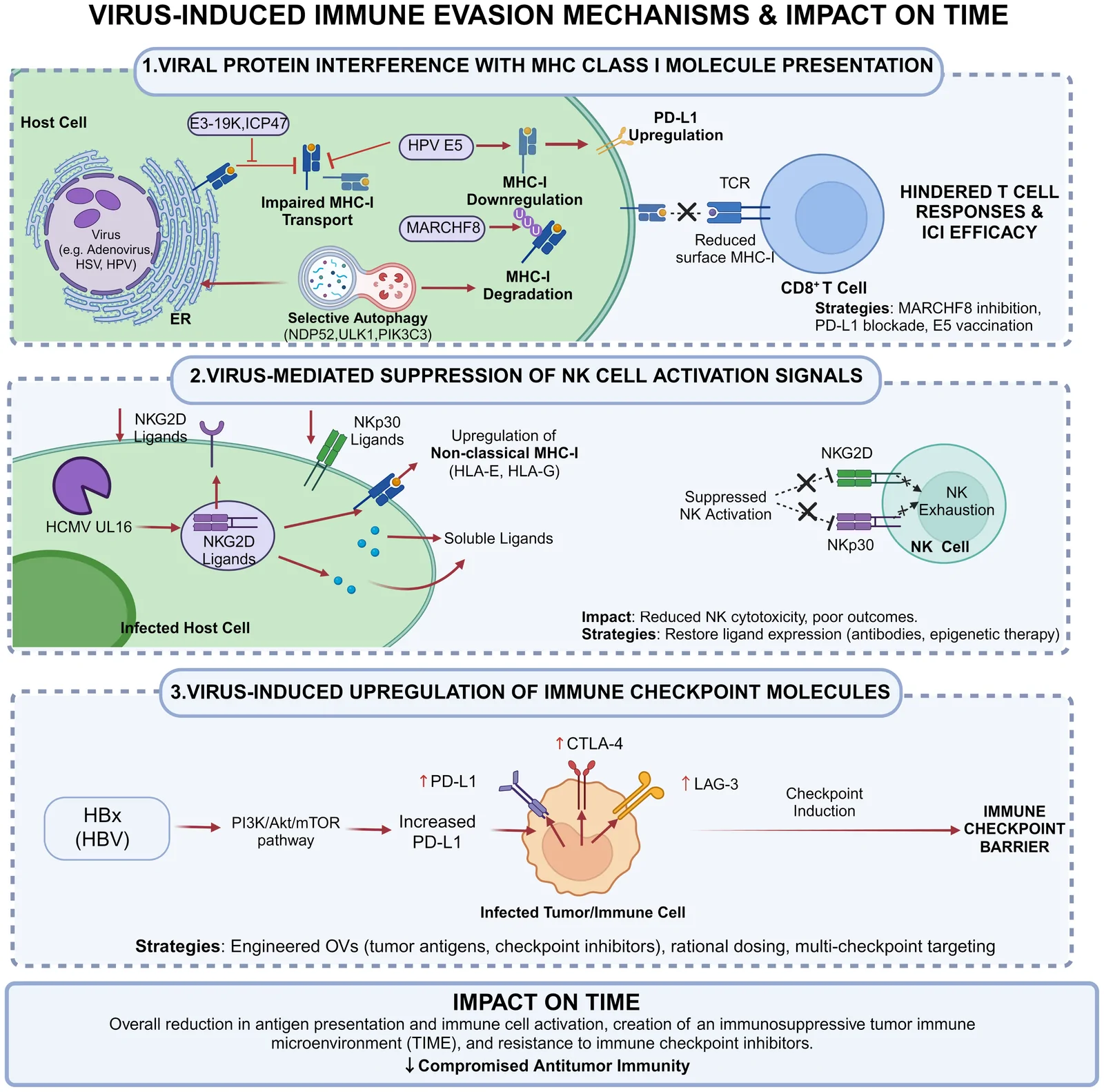
Virus-induced immune evasion mechanisms in the tumor immune microenvironment. Depicts key viral immune escape strategies targeting the TIME. Mechanisms include: 1) viral proteins interfering with MHC-I antigen presentation to impair CD8+ T cell recognition, 2) suppression of NK cell activating signals via NKG2D ligand sequestration or soluble decoys, and 3) upregulation of immune checkpoint molecules (PD-L1, CTLA-4, LAG-3) to induce T cell exhaustion. The interplay between viral immune evasion and tumor progression is illustrated, emphasizing potential therapeutic interventions to restore immune surveillance. All mechanisms depicted in this figure derive from persistent infection by oncogenic viruses and not from therapeutic oncolytic viruses or viral vectors.

## 5 Virus-related antigens as targets for immunotherapy

### 5.1 Immunogenicity of viral antigens and T cell recognition

Viral antigens encoded by oncogenic viruses such as HPV, EBV, and HBV exhibit high immunogenicity, making them prime targets for immune recognition and immunotherapy. Key viral proteins like HPV E6 and E7, EBV latent membrane proteins LMP1 and LMP2, and HBV surface antigen (HBsAg) are expressed persistently in tumor cells and differ substantially from host proteins, which facilitates their presentation by MHC-I molecules to CD8+ CTLs (4).

Clinical evidence supports the therapeutic potential of targeting viral antigens. For instance, T cell receptor-engineered T cell (TCR-T) therapies directed against HPV E7 have demonstrated promising preliminary efficacy in cervical cancer patients. These engineered T cells are designed to recognize HPV E7 peptides presented by MHC-I molecules on tumor cells, leading to targeted cytotoxicity and tumor regression (121).

The immunogenic viral antigens are not only pivotal for direct T cell-mediated tumor cell killing but also influence the tumor immune microenvironment. Persistent viral antigen expression can sustain chronic immune activation, potentially leading to T cell exhaustion; however, it also provides continuous antigenic stimulation necessary for maintaining an active anti-tumor immune response. Moreover, the high immunogenicity of viral antigens facilitates the design of prophylactic and therapeutic vaccines. For example, multi-epitope vaccines incorporating HPV E5, E6, and E7 oncoproteins delivered via virus-like particles have elicited robust cytotoxic T lymphocyte responses and suppressed tumor growth in preclinical models, highlighting the translational potential of targeting viral antigens (122).

EBV latent proteins such as LMP1 and LMP2 are consistently expressed in EBV-associated malignancies like nasopharyngeal carcinoma and certain lymphomas, serving as targets for T cell recognition. HBsAg is similarly expressed in hepatocellular carcinoma cells arising from chronic HBV infection and can be presented to CD8+ T cells, although the immune response is often impaired due to viral immune evasion strategies and chronic infection-induced immunosuppression (123).

### 5.2 Design and optimization of viral vector vaccines

Common viral vectors such as adenoviruses, poxviruses (including Modified Vaccinia Ankara, MVA), and lentiviruses have been extensively engineered to express tumor-associated antigens (TAAs) or viral oncoproteins, thereby eliciting potent T cell and B cell responses critical for tumor control. For example, adenoviral vectors can be modified to be replication-deficient, enhancing safety while maintaining immunogenicity, and have been used to deliver antigens such as the latent membrane protein 2 (LMP2) of EBV, which is implicated in EBV-associated nasopharyngeal carcinoma (NPC). In a phase I clinical trial, an adenovirus vector expressing LMP2 successfully induced specific T cell responses in patients, demonstrating the translational potential of viral vector vaccines in targeting virus-associated tumors (34).

Optimization strategies for viral vector vaccines focus on enhancing immunogenicity, overcoming pre-existing immunity to viral vectors, and improving antigen presentation. One approach involves the use of chimeric or heterologous viral vectors to circumvent neutralizing antibodies against common vectors such as adenovirus serotype 5 (Ad5). For instance, chimeric adenoviral vectors combining Ad5 capsid with fibers from rare serotypes like Ad35 (Ad5.F35) have shown resistance to neutralization by pre-existing antibodies, thereby eliciting stronger antitumor immune responses in colorectal cancer models (32). Studies have demonstrated that priming with a lentiviral vector followed by boosting with adenoviral or self-replicating RNA vectors significantly enhances tumor antigen-specific effector and memory CD8+ T cell responses compared to homologous boosting, resulting in improved tumor protection in murine models (37).

Toll-like receptor (TLR) agonists, such as TLR7/8 agonists, have been used as adjuvants to stimulate innate immunity and enhance antigen-specific T cell responses. A recent study employing a heterologous prime-boost vaccination with a self-assembling peptide nanoparticle TLR7/8 agonist vaccine prime followed by a chimpanzee adenovirus (ChAdOx1) boost demonstrated potent CD8+ T cell induction and tumor regression in murine models, highlighting the synergistic effect of adjuvants and viral vectors (33). Moreover, the route of administration influences vaccine efficacy; intravenous boosting with adenoviral vectors has been shown to induce higher magnitude antigen-specific CD8+ T cell responses and modulate the TIME by reducing immunosuppressive monocytes and activating cross-presenting dendritic cells, thereby enhancing antitumor immunity (38).

In addition to vector and adjuvant modifications, the design of personalized viral vector vaccines targeting patient-specific neoantigens is gaining momentum. A phase Ib trial of NOUS-PEV, a personalized vaccine expressing 60 neoantigens delivered via a Great Ape Adenoviral vector prime followed by MVA boosts, demonstrated safety and immunogenicity in metastatic melanoma patients, with evidence of neoantigen-specific T cell infiltration into tumors (35).

OVs are frequently discussed alongside viral vector vaccines, but the two occupy distinct functional and hierarchical categories and are kept separate here. A viral vector vaccine is a replication-deficient or replication-attenuated delivery vehicle whose therapeutic effect depends on the antigen it encodes. An OV is a replication-competent agent whose effect begins with lysis of the infected tumor cell and with *in situ* release of that tumor’s own antigens, so that it acts as antigen source and adjuvant simultaneously. The two overlap only in that an OV can additionally be armed with a defined antigen, at which point it performs a vaccine function alongside oncolysis rather than becoming a subclass of vaccine vector; the constructs described next illustrate that overlap. For example, oncolytic adenoviruses coated with tumor antigen peptides (PeptiCRAd platform) have been shown to induce rapid and specific T cell responses without the need for genetic modification of the virus, facilitating personalized vaccine development (41). Additionally, oncolytic adenovirus-based nano-vaccines coated with tumor cell membranes encoding immunostimulatory ligands have demonstrated selective tumor targeting, induction of immunogenic cell death, and synergistic efficacy with immune checkpoint blockade in ovarian cancer models (42).

Furthermore, the integration of genetic adjuvants encoded within viral vectors offers a novel avenue to enhance vaccine-induced immunity. An adenovirus vector encoding an anti-CTLA-4 monoclonal antibody (Ad-9D9) co-delivered with an adenovirus-based vaccine significantly amplified cellular and humoral immune responses against viral and tumor antigens compared to protein-based adjuvants, underscoring the potential of genetic adjuvants to improve vaccine efficacy (36) (**Figure 3**).

**Figure 3:**
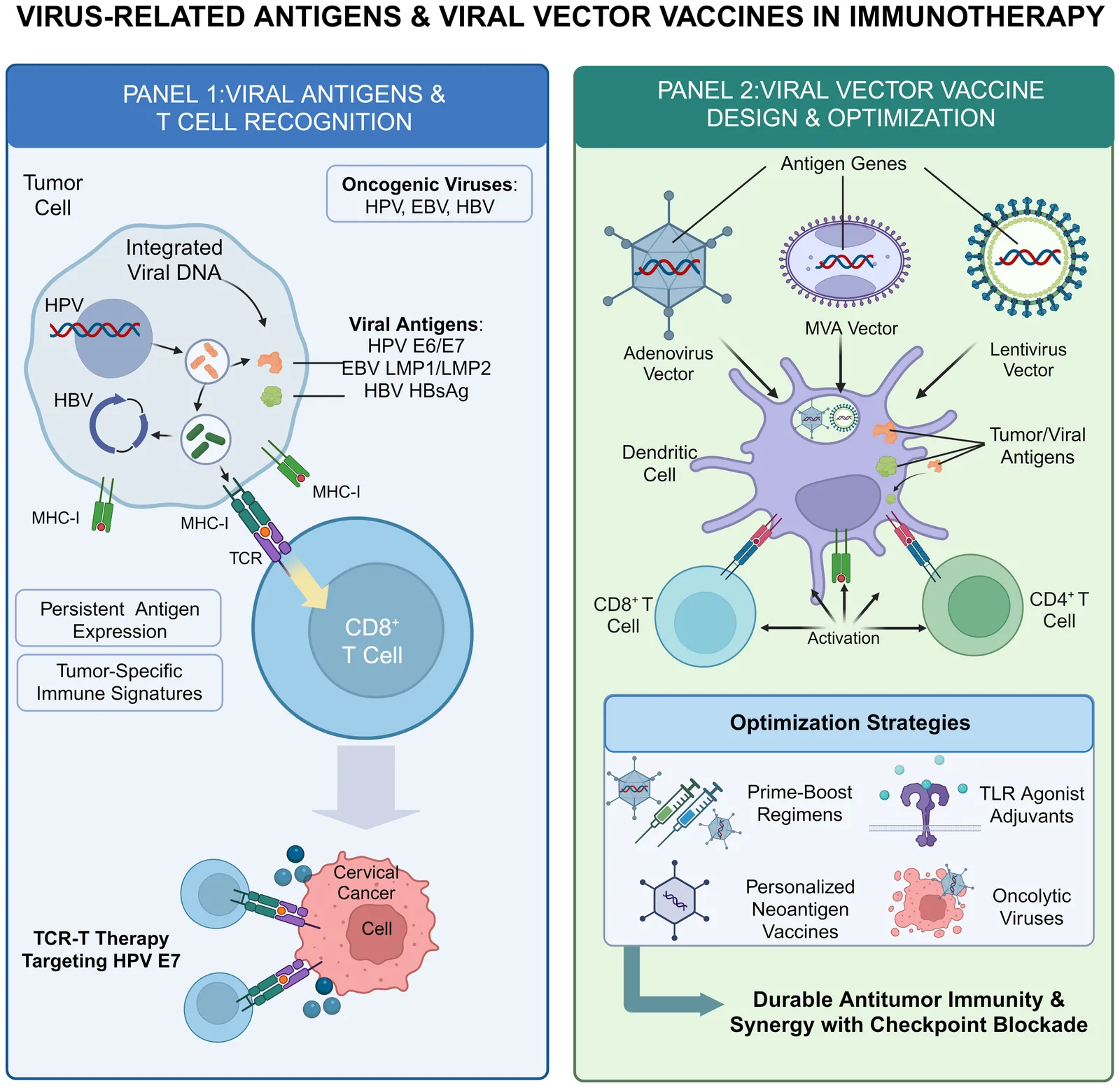
Viral antigens as targets for immunotherapy. Highlights the immunogenic viral proteins from oncogenic viruses (HPV E6/E7, EBV LMP1/LMP2, HBV HBsAg) and their recognition by CD8+ T cells via MHC-I presentation. The figure also summarizes therapeutic strategies including TCR-engineered T cells, viral vector vaccines, oncolytic viruses expressing antigens or immunomodulators, and personalized neoantigen-targeted vaccines. Synergistic immune activation, enhanced antigen presentation, and induction of durable antitumor immunity are depicted to illustrate translational applications. HPV, EBV and HBV antigens shown here derive from oncogenic viruses, whereas the delivery platforms shown (TCR-engineered T cells, viral vector vaccines, oncolytic viruses and personalized neoantigen vaccines) are therapeutic agents.

## 6 Immunoactivation effect of oncolytic viruses on TIME

### 6.1 Oncolytic virus-induced immunogenic cell death

OVs such as T-VEC and coxsackievirus induce ICD in infected tumor cells, and ICD is the central mechanism by which oncolysis is converted into adaptive antitumor immunity. Infection triggers surface exposure of calreticulin (CRT) and the release of adenosine triphosphate (ATP) and high-mobility group box 1 (HMGB1); these DAMPs act as danger signals that mature DCs and promote cross-presentation of tumor antigens to T cells, thereby bridging innate and adaptive immunity. These three molecules are not equivalent, and the distinction is mechanistically informative because each becomes available to the immune system at a different stage of cell death and by a different route. Calreticulin is translocated from the endoplasmic reticulum to the outer leaflet of the plasma membrane while the infected cell is still intact, in the pre-apoptotic phase, and remains cell-surface bound, where ecto-calreticulin acts as an “eat-me” signal that licenses uptake by dendritic cells. ATP is actively secreted through lysosomal and autophagy-dependent exocytosis early in the death process and acts at a distance, functioning as a “find-me” signal that recruits monocytes and dendritic cell precursors through purinergic receptors. HMGB1, by contrast, is a nuclear protein released passively only once membrane integrity is lost in the late apoptotic and necrotic phase, reaching the extracellular space together with genomic DNA and engaging TLR4 on myeloid cells and, through the DNA component, the cGAS-STING axis (124). A platform that kills predominantly by apoptosis therefore generates a different temporal DAMP profile from one that engages necroptosis or pyroptosis, which is one reason the immunological output of oncolysis is platform-dependent rather than generic. Vaccinia virus-based platforms engage necroptosis and pyroptosis to liberate DAMPs, with consequent DC activation and T helper 1 cytokine production essential for effective CTL responses (125), and OV infection also alters the extracellular vesicle output of tumor cells, enriching it for pathogen- and damage-associated molecular patterns that reprogram macrophages towards a proinflammatory M1 phenotype (126). Because this same DAMP repertoire underlies the synergy with checkpoint blockade discussed in Section 8.1, it is described in full here and cross-referenced rather than repeated there.

The accompanying release of type I interferons and chemokines establishes a local inflammatory milieu that functions as an *in situ* vaccine, converting an immunologically “cold” TIME into a “hot” one and enabling systemic responses against both injected and distant lesions. The effect is most pronounced in tumors with low baseline infiltration, where OV-induced antigen release and immune cell recruitment sensitize the tumor to PD-1 blockade (1). Fusogenic oncolytic vaccinia viruses, for example, enhance ICD, reduce immunosuppressive cell populations and generate durable immune memory when combined with ICIs (127), and combination with stereotactic body radiotherapy further potentiates necroptosis and DAMP release (11). Arming OVs with IL-7, IL-12, IL-23 or IL-9 has been used to sustain and amplify the resulting immune activation (128–130).

Important limitations remain, and the countermeasures developed so far are construct-specific. Antiviral immune clearance and the immunosuppressive TIME both curtail ICD-driven immunity. OVs armed with checkpoint-blocking antibodies such as anti-TIGIT, or with TGF-β inhibitors, counteract tumor-mediated immunosuppression and enhance ICD-mediated antitumor effects (131, 132), while display of the complement regulator CD55 on the virion evades neutralizing antibodies and prolongs viral persistence *in vivo (*47). ICD is therefore best regarded not as a uniform property of OVs but as an outcome whose magnitude depends on the platform, the cell death pathway it engages and the immune status of the tumor it enters.

### 6.2 Disruption of immunosuppressive networks in the TIME by oncolytic viruses

OVs have emerged as a multifaceted therapeutic modality capable of directly lysing tumor cells and profoundly remodeling the TIME to overcome immunosuppression. For instance, treatment with oncolytic herpes simplex virus type 2 (oHSV2) in murine colon carcinoma models demonstrated effective tumor elimination accompanied by a significant reduction in Tregs and MDSCs within the spleen and tumor microenvironment, while concurrently increasing effector immune populations including NK cells, CD8+ T cells, and DCs (133).

Viral infection of tumor cells often leads to downregulation of immunosuppressive cytokines such as TGF-β and IL-10, which are key mediators of immune tolerance and suppression in tumors. Simultaneously, OVs induce the upregulation of pro-inflammatory cytokines and cytotoxic mediators, including IFN-γ and granzyme B, which enhance the cytolytic activity of immune effector cells and promote antigen presentation. For example, oncolytic HSV therapies have been shown to increase IFN-γ production and granzyme B expression in tumor-infiltrating lymphocytes, thereby fostering a more immunostimulatory environment conducive to tumor clearance (133).

In pancreatic ductal adenocarcinoma (PDAC) models, oncolytic virus infection triggered a monocyte-cancer-associated fibroblast (CAF) axis where monocyte-derived interleukin-1β (IL-1β) stimulated CAFs to produce IL-6, creating an IL-6-rich milieu that impaired dendritic cell maturation and T cell activation, thereby limiting the efficacy of OV therapy (106). To address this, engineering strategies have been employed to optimize OVs by deleting viral homologs of IL-6 or incorporating transgenes that neutralize IL-6 signaling, thereby mitigating the recruitment of MDSCs and enhancing antitumor immunity. For instance, OVs armed with immune-stimulatory molecules such as OX40 ligand (OX40L) have demonstrated the ability to reprogram macrophages and neutrophils towards a pro-inflammatory phenotype while reducing regulatory T cells and MDSCs, effectively remodeling the TIME to favor immune activation (20).

Moreover, genetic modifications of OVs using advanced techniques like CRISPR/Cas9 and cre-loxp systems have enabled the development of next-generation viruses that express bispecific T cell engagers (BiTEs) or immune checkpoint inhibitors locally within the tumor. These engineered OVs not only lyse tumor cells but also enhance T cell infiltration and effector function while decreasing immunosuppressive populations such as FOXP3+ Tregs and CD206+ M2 macrophages (21).

The antiviral innate immune response, including pathways mediated by protein kinase R (PKR), can paradoxically induce TGF-β signaling, which suppresses adaptive immunity and limits OV replication and efficacy (110). Strategies to inhibit such pathways or engineer OVs to evade antiviral restriction factors have shown promise in enhancing viral spread and antitumor immunity.

Senescent cells constitute a further obstacle within these same networks, and one that has only recently been connected to virotherapy. Oncolysis, and the therapies commonly given alongside it, drive a fraction of tumor cells into senescence rather than death, and senescent cells are comparatively refractory to infection: radiation-induced senescent glioblastoma cells supported markedly less vaccinia virus infection and replication than their non-senescent counterparts, an antiviral state attributable to NF-κB-dependent secretion (134). The interleukin-8-dependent tumor self-rampart described in Section 3.2, a concentric barrier of senescent and fibrotic cells that spatially confines viral spread (105), is the same problem expressed at the level of tissue architecture. Together these observations argue that the senescent compartment should be treated as a target rather than as a by-product. Direct support comes from an engineered oncolytic HSV-1 that blocks JAG1-mediated Notch signaling: it drove glioma cells into a senescence-associated secretory phenotype, activated macrophages, and rendered the tumor susceptible to cetuximab-mediated senolysis, improving efficacy in athymic and humanized mouse models (135). Systematic review of murine data likewise indicates that inducing senescence and then clearing it controls tumor growth better than either maneuver alone (136). Whether pharmacological senolytics can be scheduled around an oncolytic virus without also removing the senescence-associated secretory signals that recruit immune cells is unresolved, and it is a question of timing rather than of principle.

## 7 Delivery and application of viral vectors in immunotherapy

### 7.1 Viral vector delivery of immune checkpoint inhibitors

The utilization of viral vectors, particularly adenoviruses and lentiviruses, to deliver genes encoding ICIs such as anti-PD-1 and anti-CTLA-4 antibodies directly into tumors represents a promising strategy to achieve localized high concentrations of these therapeutic agents while minimizing systemic exposure and associated immune-related adverse events (irAEs). This approach, often termed “*in situ* immunotherapy,” leverages the natural tumor tropism and gene delivery capabilities of viral vectors to transform the TIME into an immunologically active site, enhancing antitumor immunity.

Adenoviral vectors have been extensively studied due to their high transduction efficiency, large transgene capacity, and ability to induce robust immune responses. For example, the oncolytic adenovirus TILT-123, engineered to express cytokines such as tumor necrosis factor alpha and interleukin-2, has demonstrated safety and efficacy in preclinical models and is currently under clinical investigation in combination with anti-PD-1 therapy for melanoma patients (22). Similarly, conditionally replicative adenoviruses armed with anti-PD-1 single-domain antibodies (sdAb) have shown selective replication in osteosarcoma cells, leading to efficient tumor cell lysis and local production of functional anti-PD-1 antibodies, thereby enhancing antitumor immune responses (29).

The oncolytic HSV G47Δ combined with systemic anti-CTLA-4 antibody administration synergistically enhanced antitumor activity in murine tumor models by recruiting effector T cells and decreasing regulatory T cells within the tumor, thereby converting immune-insusceptible tumors into immune-susceptible ones (27). Moreover, HSV vectors co-expressing anti-PD-1 antibodies and interleukin-12 have demonstrated superior antitumor efficacy by modulating the TIME and enhancing T cell infiltration and activation (31).

Lentiviral vectors have been employed to deliver ICIs and tumor antigens, facilitating robust T cell responses. A lentiviral vector encoding a humanized single-chain variable fragment (scFv) against PD-1 (YST-OVH) demonstrated potent T cell activation and systemic antitumor immunity in humanized PD-1 mouse models, with enhanced efficacy when combined with anti-CTLA-4 or anti-TIM-3 antibodies (28).

The strategy of intratumoral injection of viral vectors encoding ICIs offers several advantages. For instance, intratumoral administration of a chimpanzee adenoviral vector expressing full-length anti-PD-1 monoclonal antibody resulted in significant tumor suppression and prolonged survival in a human PD-1 knockin mouse model, outperforming unarmed viruses and systemic antibody administration (25). Similarly, oncolytic vaccinia viruses engineered to express cytokines and ICIs have demonstrated the ability to remodel the immunosuppressive TIME, increase infiltration of CD8+ T cells and macrophages, and synergize with PD-1 blockade to achieve complete tumor regression in pancreatic cancer models (137).

When armed with genes encoding ICIs, OVs can further enhance antitumor immunity. For example, oncolytic vaccinia virus TG6050, encoding interleukin-12 and anti-CTLA-4 antibody, induced profound immune remodeling of the TIME, increasing interferon-gamma production, CD8+ T cell infiltration, and M1/M2 macrophage ratio while decreasing regulatory T cells, leading to strong antitumor activity in both “hot” and “cold” tumor models (26).

Preclinical studies in murine models have also explored the combination of viral vector-mediated ICI delivery with systemic ICIs or other immunotherapies. For instance, the combination of oncolytic herpes simplex virus expressing anti-PD-1 antibody and interleukin-12 showed enhanced antitumor efficacy compared to monotherapies, suggesting that viral vector-mediated local ICI expression can potentiate systemic immune responses (31). Moreover, oncolytic measles virus vectors encoding PD-1 and PD-L1 blocking antibodies elicited tumor-specific T cell memory and modest survival benefits in murine models, supporting the development of such vectors as therapeutics to induce durable antitumor immunity (138).

Safety considerations are paramount in developing viral vector-based ICI delivery systems. Similarly, the oncolytic herpes simplex virus vector YST-OVH expressing PD-1 scFv was well tolerated in nonhuman primates without neurotoxicity; no clinical safety data are yet available for this construct, so its tolerability in patients remains untested (28).

### 7.2 Viral vector delivery of cytokines and costimulatory molecules

Cytokines such as IL-12, IL-15, and GM-CSF are frequently encoded by viral vectors to create a pro-inflammatory milieu within tumors, enhancing the activation and proliferation of immune effector cells. This strategy effectively converts the immunosuppressive TIME into a more immunostimulatory environment, augmenting the infiltration and cytotoxic function of immune cells against tumor cells (22, 139).

A prime example of this approach is T-VEC, the GM-CSF-encoding oncolytic HSV-1 introduced above. T-VEC has received FDA approval for the treatment of advanced melanoma, marking a milestone in oncolytic virotherapy. Its mechanism involves selective replication within tumor cells, leading to oncolysis and release of tumor-associated antigens, while the expressed GM-CSF recruits and activates DCs at the tumor site. This dual action promotes robust T-cell priming and systemic antitumor immune responses. Clinical trials have demonstrated that T-VEC not only induces local tumor regression but also elicits abscopal effects on distant metastases (13), highlighting the capacity of viral vector-delivered cytokines to modulate the TIME effectively. The success of T-VEC underscores the therapeutic potential of viral vectors as gene delivery platforms for cytokines in cancer immunotherapy (3, 140).

Beyond cytokines, recent research has explored viral vectors engineered to deliver genes encoding costimulatory molecules such as 4-1BB ligand (4-1BBL) and OX40L. Viral vectors encoding these ligands have been shown to improve T-cell persistence and the generation of memory T cells within the tumor microenvironment, addressing a key challenge in immunotherapy where T-cell dysfunction and exhaustion limit efficacy (20).

Because the underlying gene delivery logic is not restricted to viral vectors, non-viral polymeric nanoparticles co-delivering IL-12 with 4-1BBL or OX40L have been used to test the same combinatorial principle. These formulations skew the TIME towards a proinflammatory state, increase the density and spatial proximity of cytotoxic T cells and antigen-presenting cells, and induce MHC class I and class II expression on tumor cells, thereby facilitating immune synapse formation and tumor cell killing. They offer lower immunogenicity than viral vectors but lack oncolytic activity, and they are cited here as mechanistic comparators rather than as virus-based therapies (30).

## 8 Combined therapeutic strategies of viruses and immune checkpoint inhibitors

### 8.1 Synergistic mechanisms of oncolytic virus combined with PD-1/PD-L1 inhibitors

OVs, particularly T-VEC, have emerged as promising agents in cancer immunotherapy due to their dual capacity to directly lyse tumor cells and stimulate systemic antitumor immune responses (7, 140). The clinical evidence base is dominated by T-VEC and by a small number of other platforms, and the generalizations that follow should not be extended to OVs as a class (8). A key mechanism underlying their synergy with PD-1/PD-L1 ICIs involves the induction of ICD and type I interferon (IFN) signaling, which collectively enhance tumor immunogenicity and modulate the TIME. Upon intratumoral injection, OVs selectively infect and replicate within tumor cells, leading to cell lysis and the release of tumor-associated antigens alongside DAMPs. This process triggers ICD (described in full in Section 6.1), which recruits and activate DCs, thereby promoting antigen presentation and priming of tumor-specific T cells. Concurrently, OV infection induces robust type I IFN responses that further enhance DC maturation and cross-priming of CTLs. Importantly, these inflammatory signals upregulate PD-L1 expression on tumor cells and infiltrating immune cells, creating a rational target for PD-1/PD-L1 blockade (13). This OV-mediated upregulation of PD-L1 effectively sensitizes tumors to ICIs by providing a targetable immune checkpoint, which may be otherwise absent or low in “cold” tumors with limited immune infiltration, at least in the platforms and models in which this has been tested.

Clinical and translational studies have demonstrated that T-VEC treatment increases CD8+ T cell infiltration and enhances expression of activation markers such as granzyme B and checkpoint molecules (PD-1, PD-L1, CTLA-4) in non-injected lesions, indicating systemic immune activation and remodeling of the TIME favorable for checkpoint inhibition (13). Preclinical and early clinical data suggest that OV-induced immune activation can overcome primary resistance to ICIs by converting immunologically “cold” tumors into “hot” ones, thereby enhancing the efficacy of PD-1/PD-L1 blockade (141, 142).

Clinically, the combination of T-VEC with pembrolizumab has been evaluated in multiple trials, including the pivotal phase III MASTERKEY-265 study in advanced melanoma. This trial demonstrated that while the addition of T-VEC to pembrolizumab did not significantly improve progression-free survival (PFS) or overall survival (OS) compared to pembrolizumab alone, it yielded a higher objective response rate (ORR) of 48.6% versus 41.3%, and a durable response rate (DRR) of 42.2% versus 34.1%, with manageable safety profiles consistent with each agent’s known toxicities (16). Notably, earlier phase Ib studies in recurrent or metastatic HNSCC showed that T-VEC plus pembrolizumab was tolerable and elicited partial responses in a subset of patients, although efficacy was comparable to pembrolizumab monotherapy, leading to discontinuation of the phase III portion (14). In sarcomas, a phase II trial combining T-VEC with pembrolizumab reported an ORR of 30% with acceptable safety, suggesting potential benefit in this immunotherapy-resistant tumor type (15).

The MASTERKEY-265 trial’s failure to meet primary endpoints underscores the complexity of translating preclinical synergy into clinical benefit, possibly due to patient selection, tumor heterogeneity, and the immunosuppressive TIME. Ongoing research aims to optimize dosing, scheduling, and combination strategies, including next-generation OVs armed with immunostimulatory payloads and novel delivery platforms to enhance tumor targeting and immune activation (7, 8).

In sum, OVs and PD-1/PD-L1 inhibitors act on complementary steps of the same pathway: the virus supplies tumor antigen, DAMPs and type I interferon signaling and upregulates PD-L1, and checkpoint blockade removes the resulting brake on T cell effector function. Clinical data in melanoma show higher objective and durable response rates for the combination but, to date, no demonstrated survival benefit, and the evidence remains concentrated on a small number of platforms. Biomarker-guided patient selection and next-generation armed constructs will determine whether the mechanistic synergy observed preclinically can be realized clinically (8).

### 8.2 Strategies combining viral vector vaccines with CTLA-4 inhibitors

The integration of viral vector vaccines with CTLA-4 checkpoint inhibitors represents a promising strategy to amplify antitumor immune responses by leveraging complementary mechanisms of immune activation. Viral vector vaccines, particularly those based on adenoviruses, are capable of inducing broad-spectrum T cell responses by delivering tumor-associated antigens that prime and expand antigen-specific CD8+ T cells (39).

Preclinical studies in HPV-associated tumor models have demonstrated that adenoviral vector vaccines encoding HPV oncogenic proteins, when combined with CTLA-4 blockade, significantly increase the infiltration of CD8+ T cells into tumors and suppress tumor growth more effectively than either treatment alone. For instance, adenoviral vaccines encoding the HPV E7 protein induce robust E7-specific CD8+ T cell responses, including the formation of tissue-resident memory T cells that persist in tumor sites. This combinatorial approach leads to improved tumor control and long-term protection against HPV-induced carcinomas in murine models (39).

Moreover, the co-delivery of adenoviral vectors encoding anti-CTLA-4 monoclonal antibodies alongside antigen-expressing vaccines has emerged as an innovative genetic adjuvant strategy. This method ensures spatial and temporal synchronization of CTLA-4 blockade with antigen presentation, resulting in stronger cellular and humoral immune responses compared to administration of protein-based CTLA-4 inhibitors. Importantly, the adjuvant effect is dependent on co-localization of the vectors, as separate administration sites abrogate the immunostimulatory synergy. This approach has demonstrated efficacy not only in infectious disease models but also in enhancing the immunogenicity and antitumor efficacy of adenovirus-based polyepitope vaccines encoding tumor neoantigens (36).

However, the clinical translation of viral vector vaccines combined with CTLA-4 inhibitors necessitates careful consideration of potential immune-related adverse events, particularly autoimmune toxicities. CTLA-4 blockade can lead to systemic immune activation and depletion of Tregs, which, while beneficial for antitumor immunity, may precipitate immune-mediated tissue damage. Therefore, optimizing the dosing regimens and timing of administration is critical to maximize therapeutic benefit while minimizing toxicity. Preclinical data suggest that early CTLA-4 blockade post-vaccination enhances T cell priming without exacerbating adverse effects, but clinical studies are required to define safe and effective protocols (1).

In addition, the combination of viral vector vaccines with CTLA-4 inhibitors can be further potentiated by integrating other immunomodulatory agents that target different steps of the cancer immunity cycle. For example, the addition of PD-1 blockade and Treg-depleting agents alongside adenoviral neoantigen vaccines and CTLA-4 inhibitors has resulted in near-complete tumor eradication in murine models by expanding neoantigen-specific T cells, reversing T cell exhaustion, and reducing intratumoral Tregs. This multi-pronged approach exemplifies the potential of rationally designed combination immunotherapies to overcome tumor immune evasion (40) (**Figure 4**).

**Figure 4:**
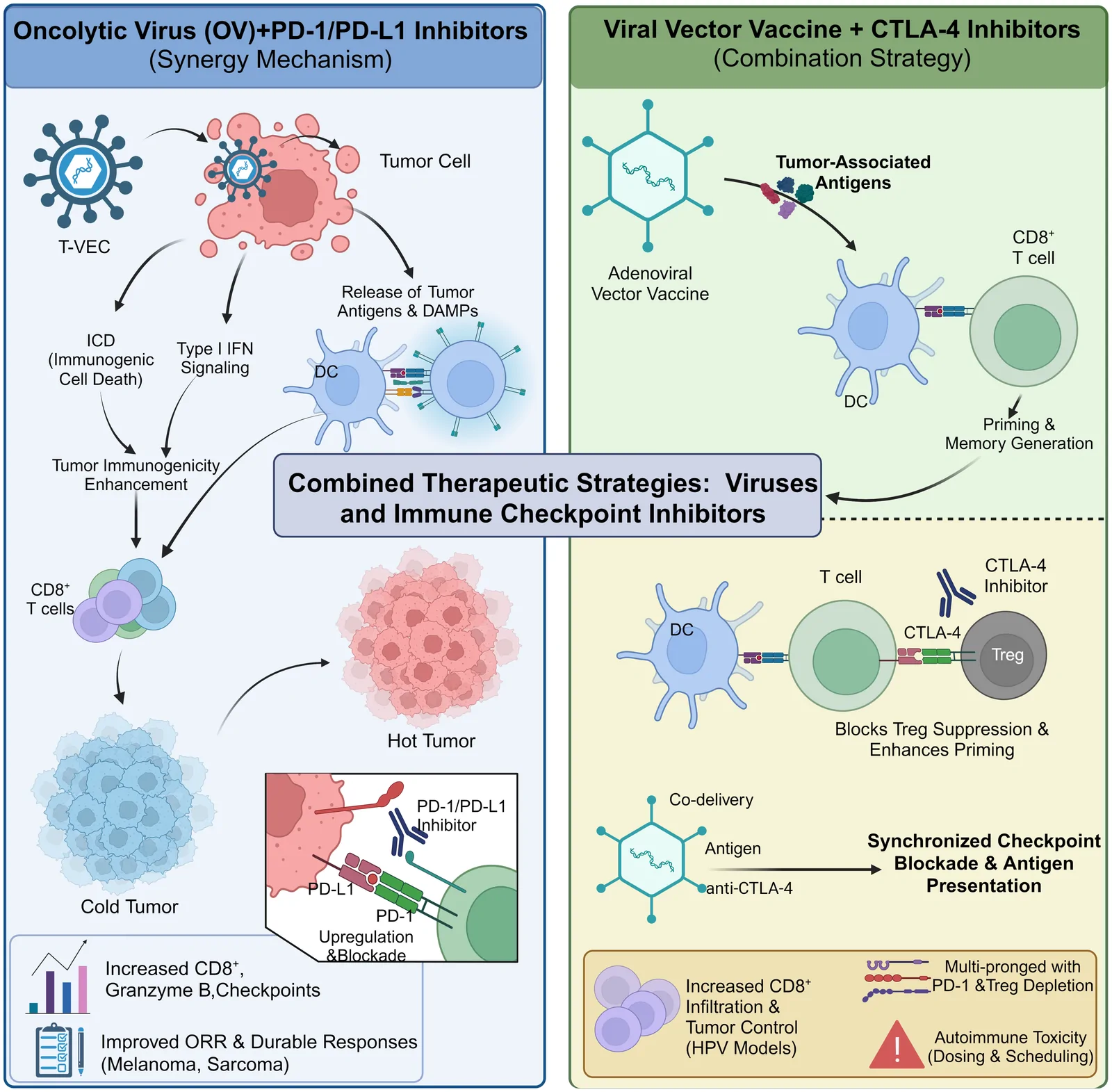
Combined therapeutic strategies of viruses and immune checkpoint inhibitors. Demonstrates the synergistic mechanisms of OVs combined with PD-1/PD-L1 or CTLA-4 inhibitors. OVs induce ICD, release DAMPs and tumor antigens, and trigger type I interferon responses, priming T cell activation. Immune checkpoint blockade releases inhibitory signals, amplifying CD8+ T cell and NK cell activity. Viral vector vaccines delivering antigens and anti-CTLA-4 antibodies enhance T cell priming, tissue-resident memory formation, and Treg depletion. The combined approach converts “cold” tumors into “hot” tumors, improving immunotherapy efficacy and systemic antitumor immunity. All viral agents depicted in this figure are therapeutic (oncolytic viruses and viral vector vaccines); no mechanism shown refers to natural infection by an oncogenic virus.

## 9 Metabolic and epigenetic regulation in virus-TIME interactions

### 9.1 Virus-induced metabolic reprogramming and its impact on the TIME

Virus-induced metabolic reprogramming influences the TIME by altering metabolic pathways in tumor cells and in infiltrating immune cells. Chronic HBV infection shifts tumor cell metabolism towards aerobic glycolysis: the HBx protein enhances glycolytic flux through NF-κB p65-dependent induction of hexokinase 2, which in turn promotes HBx-initiated hepatocellular carcinogenesis (143), and lactate accumulation is increasingly recognized as a feature of HBV-related HCC that links viral infection to a permissive metabolic environment (144). The immunological consequences are twofold. Extracellular lactate and the associated acidification directly impair CTL effector function, and lactate additionally promotes M2-like macrophage polarization and CD8+ T cell exhaustion through TGF-β/SNAIL signaling (17), while also favoring the accumulation of MDSCs. HIF-1α has been proposed as a further mediator of the glycolytic shift during HBV infection; because the primary evidence in this system centers on the NF-κB/HK2 axis, the HIF-1α limb is presented here as less firmly established rather than as an established mechanism.

In the context of OV therapy, viral replication is highly dependent on the host cell’s metabolic resources, which can exacerbate nutrient competition within the TIME. This metabolic hijacking can intensify the scarcity of nutrients such as glucose and amino acids, which are critical for the survival and function of infiltrating immune cells, including effector T cells and NK cells. Consequently, the metabolic competition induced by viral replication may impair immune cell viability and function, potentially limiting the efficacy of OV-mediated immunotherapy (18). Moreover, the metabolic stress imposed by viral infection can induce endoplasmic reticulum (ER) stress and unfolded protein response (UPR) pathways in tumor cells, further influencing immune cell recruitment and activation within the TIME (48).

The direction of this metabolic interaction differs between oncogenic and therapeutic viruses, and the difference has practical consequences. In persistent oncogenic virus infection the metabolic shift is chronic and largely tumor cell-intrinsic, and it disadvantages effector lymphocytes over a prolonged period. In virotherapy the metabolic burden is acute and localized to infected cells, but it is superimposed on a microenvironment in which glucose, glutamine and arginine are already limiting. The arginine dependency noted in Section 2.3 is a concrete illustration. It follows that metabolic interventions intended to support T cell function, such as inhibition of lactate export, buffering of extracellular pH or arginine supplementation, may simultaneously alter viral replication in either direction. Metabolic co-therapies for virotherapy therefore require explicit evaluation of both immunological and virological endpoints rather than immunological endpoints alone.

The interplay between virus-induced metabolic reprogramming and immune modulation is complex and multifaceted. For example, oncolytic herpes simplex virus type 2 (OH2) treatment in malignant ascites models induces a regional cytokine storm characterized by elevated IL-6 levels, which is closely linked to metabolic alterations within the tumor milieu. This cytokine milieu promotes infiltration and activation of CD4+ and CD8+ T cells, suggesting that viral-induced metabolic and inflammatory changes can convert an immunologically “cold” TIME into a more inflamed and immunoreactive state (12). However, the initial metabolic reprogramming may also upregulate immune checkpoint molecules such as PD-L1, contributing to adaptive immune resistance and highlighting the need for combination strategies to overcome metabolic and immunologic barriers (12).

Furthermore, the acidic microenvironment resulting from lactate accumulation due to enhanced glycolysis not only suppresses T cell function but also promotes the recruitment and polarization of immunosuppressive cells such as MDSCs and TAMs. TAMs, particularly M1-like subsets, have been implicated in mediating resistance to oncolytic virus therapies by limiting viral persistence and suppressing CD8+ T cell responses, underscoring the intricate relationship between metabolic reprogramming and immune evasion mechanisms (145).

In brain tumors such as gliomas, oncolytic adenoviruses engineered to express immune-stimulatory cytokines like IL-12 have demonstrated the ability to reprogram the TIME by promoting T cell infiltration and shifting the metabolic landscape to favor immune activation (18). Similarly, in hepatocellular carcinoma, engineered oncolytic herpes simplex viruses expressing multiple cytokines and immune checkpoint blockade fusion proteins have shown promise in reshaping the TIME and overcoming resistance to systemic therapies, partly through modulation of tumor metabolism and immune cell function (146).

### 9.2 Regulation of epigenetic modifications in the TIME by viruses

Viruses manipulate the epigenetic landscape of host cells and thereby modulate the TIME in ways that affect immune surveillance and therapeutic responsiveness. The evidence is strongest for the γ-herpesviruses and for HPV. EBV nuclear antigen 3A inactivates intergenic enhancers and establishes polycomb-associated repressive signatures across the chromatin domain encoding the T cell-attracting chemokines CXCL9 and CXCL10, directly linking an EBV latent protein to reduced effector cell recruitment (19); EBV nuclear antigen 3C recruits histone deacetylase activity and associates with the corepressors mSin3A and NCoR, providing an enzymatic basis for such repression (147). In HPV-positive cells, E7 represses components of the antigen processing and presentation pathway and lowers surface MHC-I, with the functional consequence that infected cells become less visible to CD8+ T cells while becoming more susceptible to NK cell killing (148). The proposal that E7 acts principally through direct recruitment of HDACs to the MHC-I promoter is less well substantiated than the downstream phenotype, and the phenotype rather than a single mechanism is therefore stated here.

In EBV-associated gastric carcinoma, viral infection induces extensive aberrant promoter hypermethylation, including hypermethylation of the interferon regulatory factor 5 promoter, which silences an interferon pathway regulator with tumor-suppressive and innate immune functions (149). The resulting attenuation of interferon-responsive gene expression weakens antiviral and antitumor immunity and fosters an immunosuppressive milieu that compromises the recruitment and activation of effector immune cells. This is described as hypermethylation of interferon pathway and immune-related loci rather than as silencing of IFN-γ signaling as such, because the targets mapped in this tumor type are interferon regulators rather than components proximal to the IFN-γ receptor.

Therapeutically, epigenetic drugs such as HDAC inhibitors have demonstrated the capacity to reverse virus-induced immune suppression by restoring the expression of silenced immune genes and enhancing antigen presentation. When combined with oncolytic viruses, which themselves can induce immunogenic cell death and stimulate antitumor immunity, HDAC inhibitors exhibit synergistic potential. This combination can potentiate the infiltration and activation of cytotoxic T cells and NK cells within the TIME, overcoming viral immune evasion strategies. Moreover, epigenetic modulation may sensitize tumors to immune checkpoint blockade by upregulating PD-L1 expression and other immune regulatory molecules, thereby enhancing the efficacy of immunotherapies. Collectively, these findings highlight the critical interplay between viral oncogenes, epigenetic modifications, and immune regulation in the TIME, offering promising avenues for combinatorial therapeutic strategies that integrate epigenetic drugs with oncolytic virotherapy to improve cancer immunotherapy outcomes (150, 151). In direct support of this combination, the pan-HDAC inhibitor panobinostat improved the therapeutic efficacy of oncolytic herpes simplex virus combined with PD-1/PD-L1 blockade in glioma and squamous cell carcinoma models (152).

Two caveats temper the therapeutic reading of this section. First, epigenetic drugs act on host immune cells as well as on tumor cells: HDAC inhibition can enhance oncolytic replication and antigen presentation while simultaneously dampening the type I interferon response on which OV-induced immunity depends, so the net effect is schedule-dependent and must be established empirically for each virus-drug pair (152). Second, because epigenetic modulation upregulates PD-L1 alongside the antigen processing machinery, the same intervention that improves tumor recognition can deepen checkpoint-mediated suppression. This is the mechanistic argument for the triple combinations of epigenetic modulator, OV and checkpoint inhibitor now entering preclinical evaluation, and Section 10 considers how such multi-agent regimens can be monitored and made safe.

## 10 Clinical translation challenges and future directions of virus-TIME interaction research

### 10.1 Mechanisms of resistance to viral immunotherapy

Resistance to viral immunotherapy arises at three separable levels — the infected tumor cell, the host response to the vector, and the microenvironment — and distinguishing between them is a precondition for rational biomarker and combination design rather than a matter of classification. The first level is viral entry and replication. Tumor cells may downregulate viral receptors, reducing viral entry and replication, as observed with alterations in receptor expression that limit viral tropism. Defects in interferon signaling pathways within tumor cells can impair antiviral responses, paradoxically affecting viral replication dynamics and immune activation. One practical consequence is that interferon competence predicts oncolysis and checkpoint inhibitor response in opposite directions, so a single interferon signature cannot be used to select patients for the combination without specifying which component of it the signature is meant to predict. Prior infection by an unrelated virus can also confer resistance: in models of T-cell lymphoma, previous HTLV-1 infection drives resistance to oncolytic reovirus therapy through JAK/STAT signaling, which can be antagonized pharmacologically (49). Addressing these resistance mechanisms requires multifaceted approaches, including the engineering of viruses to target alternative receptors or to evade interferon-mediated antiviral states (8).

The second level concerns the host response to the vector rather than to the tumor, and it is what currently confines most platforms to injectable lesions. Pre-existing and treatment-induced neutralizing antibodies, complement activation and clearance by the mononuclear phagocyte system all limit systemic delivery; the engineering responses include display of complement regulators such as CD55 on the virion, incorporation of CD47 extracellular domains to evade phagocytic clearance, and serotype or fiber switching to escape pre-existing anti-vector immunity (32, 46, 47). An antiviral response that develops too rapidly also truncates replication before sufficient antigen has been released, which is why dosing interval and sequence relative to checkpoint blockade are not interchangeable variables, and why repeated dosing has in some settings increased immune-mediated toxicity without adding efficacy (45).

The third level is the microenvironment, and it is the least tractable because the virus itself contributes to it. Suppressive myeloid populations recruited by virus-induced IL-6 and IL-8 restore suppression as oncolysis proceeds (105, 106), and the relationship is reciprocal: intratumoral MDSCs deplete arginine and thereby inhibit viral replication, so the suppressive compartment is at once a consequence and a cause of resistance (98). Senescent tumor cells raise a physical and antiviral barrier to viral spread (105, 134). Adaptive upregulation of PD-L1, and of TIM-3 following virus-driven dendritic cell inactivation, can convert an initially productive response into a checkpoint-limited one (88). Countermeasures under development extend beyond retargeting: viruses engineered to express immunomodulatory molecules or metabolic regulators that can reprogram the TIME to a more immunogenic state (50). Combining viral therapy with metabolic modulators or immune checkpoint inhibitors may overcome resistance by enhancing immune cell infiltration and function (153). Each of the three levels defines a measurable quantity as well as a candidate intervention, which is why resistance is set out here before biomarker development rather than after it.

### 10.2 Personalized viral immunotherapy and biomarker development

The three levels of resistance set out above define what a useful biomarker has to measure, and the candidate markers below are grouped accordingly. The heterogeneity of TIME in response to viral immunotherapy is influenced by multiple factors, including the type of virus used, the tumor histology, and the individual patient’s immune background. Viral load quantification, immune scoring of the TIME, and T-cell receptor (TCR) repertoire analysis emerge as critical parameters for biomarker development. For example, studies have demonstrated that oncolytic viruses like vaccinia and adenoviruses can modulate the TIME by inducing immunogenic cell death and promoting antigen presentation, thereby enhancing T-cell infiltration and activation (154, 155).

Advanced single-cell RNA sequencing (scRNA-seq) and spatial transcriptomics technologies have revolutionized the ability to dissect the dynamic interactions between viruses and the TIME at unprecedented resolution. For instance, scRNA-seq analyses in glioblastoma models treated with oncolytic viruses have revealed ferroptosis-related gene signatures and immune cell phenotypes that correlate with therapeutic response, highlighting potential biomarkers for patient stratification (156).

A notable example of biomarker development is the identification of IFN-γ signaling gene signatures within tumors as predictive markers for the efficacy of oncolytic virotherapy. Tumors exhibiting high baseline or induced expression of IFN-γ-responsive genes tend to respond better to oncolytic HSV-based therapies, such as T-VEC, which has been approved for melanoma treatment (157). Conversely, “cold” tumors with low IFN-γ activity may require combination approaches to sensitize the TIME, such as co-administration of immune checkpoint inhibitors or epigenetic modulators (155, 158).

### 10.3 Safety management of viral immunotherapy

Viral immunotherapy, while a promising modality for cancer treatment, presents significant safety challenges that necessitate rigorous management and monitoring systems. One of the primary safety concerns is the induction of systemic inflammatory responses, such as cytokine release syndrome (CRS), which can lead to severe clinical manifestations including fever, hypotension, and multi-organ dysfunction. Additionally, viral immunotherapy carries the risk of autoimmune toxicities, where immune responses may target normal tissues, leading to adverse events such as dermatitis, colitis, or endocrinopathies. Another critical safety issue is the potential for viral dissemination beyond the intended tumor site, which could cause off-target infections or damage to healthy tissues. The development of engineered viruses with conditional replication capabilities—such as conditionally replicative oncolytic viruses—has been a strategic advancement to enhance safety by restricting viral replication to tumor cells, thereby minimizing systemic exposure (22). Furthermore, the integration of real-time imaging and biomarker assessments allows for early detection of adverse events and timely intervention (159).

## 11 Conclusion

The interplay between viruses and the TIME represents a dynamic and multifaceted frontier in cancer immunotherapy, and its defining feature is that two categories of virus act on the same microenvironment in opposite directions. Persistent infection by oncogenic viruses builds an immunosuppressive TIME; therapeutic viruses are engineered to dismantle one. This review has traced both directions in turn, covering cellular remodeling, cytokine networks, immune evasion, viral antigens as therapeutic targets, oncolysis, vector-mediated delivery, combination with checkpoint blockade, and the metabolic and epigenetic layer that underlies all of them. The integration of the two categories, rather than either one alone, is where the practical implications lie.

Placed side by side, the two categories mirror one another. Oncogenic viruses downregulate MHC-I and sequester NK cell activating ligands, whereas OVs raise MHC-I through type I interferon and IFN-γ and release DAMPs that restore antigen presentation. Oncogenic viruses sustain IL-10, TGF-β and VEGF and expand Tregs and MDSCs, whereas OVs induce IL-12, IL-15 and type I interferon, although several OV platforms also induce IL-6 and IL-8 and thereby recruit the same suppressive populations they are intended to displace. Oncogenic viruses drive chronic antigen exposure and terminal exhaustion, whereas OVs drive acute antigen release and T cell priming, yet can also inactivate dendritic cells and deepen exhaustion under some conditions. Three therapeutic implications follow. First, in virus-associated tumors the oncogenic virus has already fixed a suppressive baseline, so virotherapy does not act on a neutral background and should be evaluated against the specific evasion program present; clonal MHC-I loss, for instance, will limit any strategy that depends on CD8+ recognition, whether or not an OV is used. Second, the viral antigens that make these tumors tractable immunotherapeutic targets are supplied by the oncogenic virus itself, so the two categories can be combined by design, with a therapeutic vector delivering the oncogenic virus’s own antigens while its replication inflames the microenvironment. Third, because the suppressive mechanisms engaged by the two categories converge on IL-6, IL-8, MDSCs and PD-L1, the same adjunctive agents may be required in both settings, which argues for evaluating virotherapy in virus-associated cancers as a distinct clinical context rather than pooling it with virus-negative disease.

Virus-associated antigens remain the most concrete opportunity identified here. They are tumor-restricted, shared between patients, and already form the basis of TCR-engineered T cell products and vaccine constructs in early-phase trials. Their value is nonetheless conditional on an intact presentation pathway, which the same viruses frequently dismantle, so antigen availability and antigen presentation have to be assessed together rather than treated as one property.

The synergistic potential of combining viral therapies with ICIs represents another critical advancement. Preclinical studies consistently show antitumor immunity amplified beyond what either modality achieves alone, whereas the randomized clinical evidence so far extends only to improved objective and durable response rates, without a demonstrated survival benefit. Factors such as treatment timing, dosing schedules, and patient selection criteria must be meticulously refined to maximize therapeutic synergy while minimizing adverse effects.

It is worth stating plainly how far this field remains from routine clinical use. One oncolytic virus has regulatory approval, in one disease, after two decades of development; the single randomized phase III test of an oncolytic virus added to checkpoint blockade improved response rates but not survival; systemic delivery is unsolved for every platform, so most agents reach only injectable lesions; no biomarker has been validated for patient selection; and the mechanisms invoked most often in support of the approach, conversion of cold tumors to hot ones among them, are demonstrated in murine models and in a small number of platforms rather than established as properties of the class. The mechanistic case assembled in this review is strong. The clinical case is not yet made, and treating the two as equivalent has been a persistent weakness of this literature.

Emerging insights into the metabolic and epigenetic regulation of the virus-TIME interface add both complexity and opportunity, and the priorities for translation follow directly from them: individualized biomarkers to identify the patients most likely to benefit and to monitor response; safety frameworks matched to the specific toxicities of replicating agents, including cytokine release and off-target viral dissemination; and mechanistic dissection of resistance, encompassing receptor loss, interferon pathway defects and compensatory expansion of suppressive myeloid populations, to inform the design of next-generation constructs.

In conclusion, virus-mediated modulation of the tumor immune microenvironment offers a coherent paradigm for cancer immunotherapy, provided that the two categories of virus are kept analytically distinct and then combined deliberately. Realizing this will require the integration of virology, immunology and oncology, together with the biomarker and safety work needed to convert platform-specific mechanisms into treatments that are precise, safe and durable for patients with virus-associated and virus-negative cancers alike.
